# Effects on childhood infections of promoting safe and hygienic complementary-food handling practices through a community-based programme: A cluster randomised controlled trial in a rural area of The Gambia

**DOI:** 10.1371/journal.pmed.1003260

**Published:** 2021-01-11

**Authors:** Semira Manaseki-Holland, Buba Manjang, Karla Hemming, James T. Martin, Christopher Bradley, Louise Jackson, Makie Taal, Om Prasad Gautam, Francesca Crowe, Bakary Sanneh, Jeroen Ensink, Tim Stokes, Sandy Cairncross

**Affiliations:** 1 Institute of Applied Health Research, College of Medical and Dental Sciences, University of Birmingham, Edgbaston, United Kingdom; 2 Directorate of Public Health and Social Welfare, Ministry of Health of the Government of Gambia, Quadrangle, Banjul, The Gambia; 3 School of Geography, Earth and Environmental Sciences, University of Birmingham, Edgbaston, United Kingdom; 4 American International University West Africa, Banjul, The Gambia; 5 WaterAid, London, United Kingdom; 6 National Public Health Laboratory Services, Ministry of Health and Social Welfare, Kotu, The Gambia; 7 London School of Hygiene & Tropical Medicine, London, United Kingdom; 8 Department of General Practice and Rural Health, Dunedin School of Medicine, University of Otago, Dunedin, New Zealand; Instituto de Investigacion Nutricional, PERU

## Abstract

**Background:**

The Gambia has high rates of under-5 mortality from diarrhoea and pneumonia, peaking during complementary-feeding age. Community-based interventions may reduce complementary-food contamination and disease rates.

**Methods and findings:**

A public health intervention using critical control points and motivational drivers, delivered February–April 2015 in The Gambia, was evaluated in a cluster randomised controlled trial at 6- and 32-month follow-up in September–October 2015 and October–December 2017, respectively. After consent for trial participation and baseline data were collected, 30 villages (clusters) were randomly assigned to intervention or control, stratified by population size and geography. The intervention included a community-wide campaign on days 1, 2, 17, and 25, a reminder visit at 5 months, plus informal community-volunteer home visits. It promoted 5 key complementary-food and 1 key drinking-water safety and hygiene behaviours through performing arts, public meetings, and certifications delivered by a team from local health and village structures to all villagers who attended the activities, to which mothers of 6- to 24-month-old children were specifically invited. Control villages received a 1-day campaign on domestic-garden water use. The background characteristics of mother and clusters (villages) were balanced between the trial arms. Outcomes were measured at 6 and 32 months in a random sample of 21–26 mothers per cluster. There were no intervention or research team visits to villages between 6 and 32 months. The primary outcome was a composite outcome of the number of times key complementary-food behaviours were observed as a proportion of the number of opportunities to perform the behaviours during the observation period at 6 months. Secondary outcomes included the rate of each recommended behaviour; microbiological growth from complementary food and drinking water (6 months only); and reported acute respiratory infections, diarrhoea, and diarrhoea hospitalisation. Analysis was by intention-to-treat analysis adjusted by clustering. (Registration: PACTR201410000859336). We found that 394/571 (69%) of mothers with complementary-feeding children in the intervention villages were actively involved in the campaign. No villages withdrew, and there were no changes in the implementation of the intervention. The intervention improved behaviour adoption significantly. For the primary outcome, the rate was 662/4,351(incidence rate [IR] = 0.15) in control villages versus 2,861/4,378 (IR = 0.65) in intervention villages (adjusted incidence rate ratio [aIRR] = 4.44, 95% CI 3.62–5.44, *p <* 0.001), and at 32 months the aIRR was 1.17 (95% CI 1.07–1.29, *p* = 0.001). Secondary health outcomes also improved with the intervention: (1) mother-reported diarrhoea at 6 months, with adjusted relative risk (aRR) = 0.39 (95% CI 0.32–0.48, *p <* 0.001), and at 32 months, with aRR = 0.68 (95% CI 0.48–0.96, *p* = 0.027); (2) mother-reported diarrhoea hospitalisation at 6 months, with aRR = 0.35 (95% CI 0.19–0.66, *p* = 0.001), and at 32 months, with aRR = 0.38 (95% CI 0.18–0.80, *p* = 0.011); and (3) mother-reported acute respiratory tract infections at 6 months, with aRR = 0.67 (95% CI 0.53–0.86, *p* = 0.001), though at 32 months improvement was not significant (*p* = 0.200). No adverse events were reported. The main limitations were that only medium to small rural villages were involved. Obtaining laboratory cultures from food at 32 months was not possible, and no stool microorganisms were investigated.

**Conclusions:**

We found that low-cost and culturally embedded behaviour change interventions were acceptable to communities and led to short- and long-term improvements in complementary-food safety and hygiene practices, and reported diarrhoea and acute respiratory tract infections.

**Trial registration:**

The trial was registered on the 17th October 2014 with the Pan African Clinical Trial Registry in South Africa with number (PACTR201410000859336) and 32-month follow-up as an amendment to the trial.

## Abstract 2

Semira Manaseki-Holland and co-workers evaluate a behaviour-change intervention aimed at improving food hygiene for infants in West Africa.

## Author summary

**Why was this study done?:**

- Children aged 0–2 years are fed complementary foods and have the highest rates of diarrhoea and food-borne illnesses. Most hygiene or child nutrition programmes do not directly address complementary-food safety and hygiene.
- We performed a large cluster randomised controlled trial (RCT) of a community-level complementary-food safety and hygiene intervention in which medium- and long-term health outcomes were measured.

**What did the researchers do and find?:**

- We used a cluster RCT study to test a novel community-level campaign-like intervention in 15 intervention and 15 control villages in rural Gambia and evaluated outcomes at 6 and 32 months post-intervention.
- Target behaviours were identified through systematic assessment of motivational drivers for behaviour change and critical control points (CCPs) for contamination in complementary-food preparation and handling practices identified through a hazard analysis assessment.
- The intervention was implemented at the community level and focussed on mothers of young children; it was delivered by a combination of performing arts, public meetings, household visits, commitment ceremonies, and certifications of mothers and communities.
- Sixty-nine percent of mothers with complementary-feeding-age children were actively involved in the campaign. At 6 months post-intervention, adoption of behaviours was high; child’s reported diarrhoea, hospital admission for diarrhoea, and respiratory disease were reduced by 60%, 60%, and 30%, respectively. At 32 months post-intervention, mothers in control villages had adopted some of the behaviours promoted by the intervention (intervention ‘cross-contamination’), but practice of the behaviours in the intervention villages remained significantly higher, with a 30% reduction in reported diarrhoea and a 40% reduction in hospital admissions for diarrhoea.
- Clinic data collected by another research team confirmed reduced diarrhoea visits.
- These outcomes were achieved without further programmatic input after 5 months. Mothers without babies at the time of the programme were subsequently engaged by community members and adopted the behaviours.
- The intervention adds further value as it was delivered by a team from the public health and village structures, and was low intensity and low cost.

**What do these findings mean?:**

- We demonstrate that these low-cost, culturally embedded interventions are acceptable, adopted, and sustained at the community level, with high levels of behaviour change and reported reduction in diarrhoea and acute respiratory infection outcomes in the short term, and significant long-term effects.
- Interventions of this form could make an invaluable contribution to diarrhoea prevention; although our findings are limited to small rural villages, these are nonetheless common in low- and middle-income countries. Our conclusions need to be tested in urban and peri-urban settings.
- To date, insufficient attention has been devoted to food safety and food hygiene practices aimed at preparing, handling, and feeding complementary food. There have also been insufficient assessments of programmes with cultural performing arts that can engage whole communities to support mothers with behaviour change.

## Introduction

Globally, 1.73 billion diarrhoea episodes and 120 million pneumonias are estimated to occur in children aged <5 years each year, resulting in approximately 2 million deaths in 2010 [1]. Enteric diseases further result in infant malnutrition [2–4]. Despite new vaccine and treatment measures [3], these diseases are an immense public health problem in low- and middle-income countries (LMICs), and are targeted by the Sustainable Development Goals (SDGs).

The complementary-food period is when a child starts eating solids but does not yet eat the family meal (usually 6 to 24 months of age). It is associated with the highest rates of diarrhoea and respiratory infections: 72% of deaths from diarrhoea and 81% from pneumonia occur in children younger than 2 years, while >50% of all diarrhoea deaths occur at 6–11 months of age, when complementary food is first introduced [1]. High rates of diarrhoea continue in children aged 12–24 months [1].

The World Health Organization (WHO) estimates that 600 million episodes of illness, 420,000 deaths, and 33 million disability-adjusted life years (DALYs) were attributable to consumption of contaminated food worldwide in 2010 (which excludes diseases related to milk, water, and other drinks) [5]. This emphasises the considerable public health impact of contaminated food. Importantly, children <5 years of age experienced 43% of the food-borne disease (FBD) burden and DALYs globally, despite representing only 9% of the global population. Regionally, Africa had the greatest burden, although the WHO estimates were conservative since they largely excluded diarrhoeal disease associated with human immunodeficiency virus (HIV) infection [5], which makes patients particularly vulnerable.

The most plausible approach to prevent diarrhoeal infection is to avoid ingestion of contaminated food and water. In contrast with the many studies assessing the effects of improved water and sanitation on diarrhoeal disease, the majority of food safety efforts have been too general to succeed in interrupting possible disease transmission pathways [6,7]. In particular, insufficient attention has been devoted to food safety and food hygiene practices aimed at preparing and handling complementary food or household food [6,7]. The WHO has called for rigorous studies and interventions to prevent complementary-food contamination [5–7], but there is a paucity of public health or community-level intervention trials with health outcomes.

We applied the findings from rapid assessment formative research in The Gambia [8,9] to a previous community-level intervention, evaluated in a before-and-after 8-cluster randomised controlled trial (RCT) in Nepal [10,11]. We developed a less intensive, low-cost, community-based behaviour change intervention for complementary-food safety and hygiene [8] delivered through local public health and community structures. We investigated the effects of this complex intervention using a cluster RCT design in rural Gambia on the following outcomes: the proportion of occasions mothers were observed to practice 5 key food-related behaviours at 6 months (primary outcome), the proportion of occasions mothers were observed to boil drinking water, microbiological contamination in complementary food and drinking water, and children’s rates of diarrhoea, diarrhoea hospital admission, and respiratory infection.

## Methods

The methods are in part previously published [8,9] and available in S1 and S2 Protocols, and summarised below. The CONSORT framework for reporting of cluster RCTs has been used [12] (S1 CONSORT Checklist).

### Design

This was a 1:1 parallel cluster RCT where the unit of randomisation was a whole village. The intervention was village-wide and targeted mothers of complementary-food-age children. The main intervention was delivered over 25 days (4 community campaign visits by an intervention team and village volunteers encouraging mothers in between campaign visits) [8] and was followed by a reminder visit to villages after 5 months. Three cross-sectional samples were taken to measure characteristics and outcomes: at baseline before randomisation and at 6- and 32-month post-intervention campaign events [8].

### Setting and participants

The study was conducted in The Gambia’s Central River Region (CRR) (S1 Box). CRR is The Gambia’s poorest region, with the highest rates of diarrhoea [13]. Villages with a population of 200–1,450 were eligible for inclusion because (1) the majority of the villages/towns in CRR are this size [14], (2) there were insufficient young children in smaller villages to provide a sufficient sample size (of >20 mothers), and (3) larger villages would have required the inclusion of additional public health officers in the team. All villages were Primary Health Care Programme (PHC) villages and had a male village health worker (VHW) and a traditional birth attendant (TBA), both of whom had completed a 4-week Gambian Ministry of Health (MoH) training programme. At the time of the study, the MoH was in the process of extending this programme to all villages nationally, and such community volunteers are common in sub-Saharan rural settings. Therefore, these inclusion criteria should ensure generalisability of our intervention. It was anticipated that TBAs could also act as our project volunteers and together with VHWs could informally encourage community participation (in practice, when village elders were asked to appoint women volunteers, in most cases other women were nominated and not the TBAs, but still the TBAs and VHWs, amongst other prominent villagers, were engaged and assisted the programme—see Discussion). Villages within 5 km of already selected villages, villages involved in the formative research, and the pilot villages were excluded to prevent cross-contamination.

Household inclusion criteria for the baseline and the first cross-sectional follow-up were mothers with children aged 6–24 months. The total number of children in the village was determined at baseline through census-like household visits (November–December 2014). At 6 and 32 months, the list was updated using lists maintained by VHWs and maternal and child health clinics (during September–October 2015 and October–December 2017, respectively). Non-residents and those expecting to leave the village within 6 months of the study were excluded at baseline, and households absent during the period of intervention were excluded from the sampling frame for follow-up. Mothers of children aged 6–36 months were eligible for inclusion in the 32-month follow-up (no exclusion criteria). This meant that mothers with children younger than 26 months had yet to give birth during the intervention team’s 25-day village campaign and the fifth (5-month reminder) community visit (hereafter termed ‘new mothers’). Children 26–32 months of age at 32-month follow-up were <6 months old and therefore not officially complementary-food age during the campaign and reminder visit.

### Randomisation and masking

In November–December 2014, 30 of 55 eligible clusters were selected at random, heads of villages were visited, consent obtained, and baseline data collected. Thereafter, 15 clusters were randomly allocated to each arm within strata (by north or south of the Gambia River and by quartiles of village population size) using a computerised random number generator [8]. Children were randomly selected at 6 and 32 months from the list of all village children in the chosen cluster using a random number list generated in Excel by a statistician (KH).

Due to the nature of the intervention, blinding of mothers and the intervention team was not possible. However, mothers and data collectors were masked to the assessment of the outcomes as follows: At both the 6- and 32-month assessments, mothers were informed that the assessment was investigating domestic water and food usage by observing their daily activities. New data collectors were independently recruited in each round and were unaware of the existence of the intervention or the inter-village comparison. Complementary-food safety and hygiene components of the assessment tools were concealed in a larger assessment, with a package of observation tools and questionnaires about household food and water use, mother and child activities (including observation and a questionnaire on child care and play activities), health-seeking, food for family and baby, water and sanitation, income in the household, and village activities. Thus, the data collectors were trained for, and mothers consented to, the conduct of this larger assessment of the household’s food and water consumption, health, and child care (all measures taken to reduce reactivity and observation bias are summarised in S2 Box).

The laboratory technicians were masked as all the food and water samples were labelled with codes. Data analysts were also masked.

### Intervention

The behaviour change intervention was theoretically based [8]. The main theoretical basis for the intervention was (1) psychological and motivational theories for hygienic behaviour change [15,16] aiming to influence individual and community social norms and (2) identification of corrective behaviours through the Hazard Analysis Critical Control Point (HACCP) system [17,18]. In HACCP, a critical control point (CCP) is a step or procedure at which a significant hazard occurs in food processing or handling, and at which control or corrective measures can be applied to prevent or minimise the hazard [17]. The corrective measures and motivators were identified during rapid assessment formative research [8,9] and delivered through a complex campaign-like community intervention. These measures and motivators were informed by, and included components from, community intervention studies on complementary-food safety and hygiene in Nepal [10,11] and handwashing in India [19], and were adapted to the local context after formative research [8,9]. The complex community intervention, if successful, was envisaged to enable a degree of shift to the social norm at the community level that would enable long-term behaviour change in villagers. A detailed description is provided elsewhere [8,9] and summarised in S3 Box, S1 and S2 Tables.

The main intervention was delivered to the 15 villages from February to April 2015, and in each village over 25 days (4 community campaign visits by 3 local health promotion/public health officers and 2 dramatic artists on days 1, 2, 17 and 25, and trained older mother village volunteers, the MaaSupervisors, who encouraged the mothers to follow best food safety and hygiene practice in between campaign visits) [8], hereafter called the 25-day village campaign. Approximately 5 months after the 25-day village campaign (in July–August 2015) during the rainy season, which is a busy period in village life when villagers might struggle to persist with the key behaviours, a visit by the intervention team sought to remind villagers about the behaviours. During the fourth and fifth (5-month reminder) campaign visits, communities were encouraged to continue the behaviours and disseminate them among ‘new mothers’ with no incentive or contact from outside their community.

Control villages received a 1-day health education campaign visit from a public health officer on water use in domestic vegetable gardening, including a whole community meeting. The intervention and control village activities were delivered in parallel in February–April 2015 (the dry season). There was no further contact with the villages by intervention or assessment teams after the 6-month follow-up until the 32-month follow-up.

### Outcomes

Assessments were conducted 6 and 32 months after the 25-day village campaign. The primary outcome was the difference between the intervention and control clusters at 6 months in the number of times 5 key complementary-food-related behaviours (defined in S3 Table) were observed as a proportion of the number of opportunities to perform the behaviours during the observation period. As an example of an opportunity, every time a mother started cooking/preparing food for a child during the observation period, she should have washed her hands with soap first; did she do so or not? The mother’s behaviour when starting to cook baby food is an opportunity to demonstrate the practice of washing hands with soap before cooking. Secondary outcomes are listed and defined in S3 Table. Boiling water, which was also promoted, was measured as a secondary outcome, since complementary-food safety and hygiene was the main focus of the intervention.

### Data collection during home visits

At baseline, a trained researcher visited a random selection of village households with children aged 6–24 months and completed a short questionnaire on background characteristics of families plus reported diarrhoea and acute respiratory infection (ARI) in the past 7 days. This survey took less than 15 minutes per household.

Assessments were 6 and 32 months after the 25-day village campaign, September–October 2015 (rainy season) and October–December 2017 (dry season), respectively. Further interim surveys were not conducted in order to reduce assessment rounds that might increase reactivity bias in mothers [19]. After the 5-month reminder visit by the intervention team and the 6-month follow-up by an independent assessment team, there were no further contacts with the villages until the 32-month follow-up.

At the 6- and 32-month assessments, the families were not notified of the impending assessment visit. Village heads provided written (informed) permission for all study activities before randomisation at baseline. The mothers provided written informed consent the evening before the observation days at 6 and 32 months. The next day, the female data collector arrived in the household as the mother was beginning her day making breakfast (typically between 6:30 and 7:30 AM). Methodology and tools for data collection at both the 6- and 32-month assessments were similar and were adapted from the Nepal study [10,11]. Separate groups of 12th-grade-graduate female data collectors from outside the study communities were recruited through secondary schools in CRR, and each was trained for approximately 3.5 weeks. They visited 1 village per day as a team and were each assigned to a mother. They completed a structured observation checklist from 6:30 AM to 3:00 PM, when mothers prepared the complementary food at breakfast and lunch and fed the child. All opportunities to perform the 5 key behaviours and boil water were recorded.

A socio-demographic questionnaire used at the end of the observations included sections on economic evaluation; health education; incidence of diarrhoea, ARI, and hospitalisation for the child; and other questions for diversion and nested studies. Such components included a set of questions on health facility utilisation and other health economic issues, child activity and play, health and nutrition education, and influences on the mother.

At 6-month follow-up, data collectors collected 2 samples of complementary food aseptically [20]: 1 immediately after the morning preparation before feeding the child, and 1 after storage of the food made in the morning before feeding at lunch time. Water prepared for the child was also sampled [20]. Samples were cultured for *Escherichia coli* coliforms in the Bansang District Hospital microbiology laboratory by trained study laboratory staff using established protocols [20]. The methods have been elaborated in S4 Box.

At 6- and 32-month follow-up, data collectors and families were masked to the existence of a trial and of a comparison between the arms as explained in the ‘Randomisation and masking’ section and S1 Box.

### Sample size

Formative research indicated that the population proportion of events displaying recommended handwashing behaviours was 17/150 (11.3%) [8,9]. The sample size was calculated to detect a 25% increase in behaviours in the intervention over the control arm at 6 months with 95% power, a 2-sided alpha of 0.05, an intra-cluster correlation coefficient (ICC) of 0.04 [19], and a coefficient of variation of cluster size of 0.22. With 15 clusters per arm, at least 12 mothers per cluster were required [21]. To account for possible drop-outs during the 9-hour home visit, 20 mothers were randomly selected from each village at 6 months. The study was robust to changes to the ICC, and would have over 80% power for all likely values of the ICC (0.01 to 0.1). At 32 months, 24–26 mothers were randomly selected per stratified village, aiming to recruit 10–12 mothers with children ≥26 months of age and 12–16 with children 6–25 months of age (‘new mothers’). No interim analysis was conducted.

### Statistical analysis

Data were entered and checked in an Excel database. All analyses were performed using Stata version 14. For all outcomes, the analysis was by intention to treat. As randomisation was conducted at the village level, a mixed-effect model allowed clustering within villages for all models that follow. Three models were run for each outcome: an unadjusted model, a partially adjusted model for village-level stratification covariates specified for the primary outcome analysis (north/south of river and village size), and a fully adjusted model with further adjustment for pre-specified covariates (mother’s age and education, sex of child, and number of children in household).

Count outcomes (e.g., behaviours) were analysed using a mixed-effect Poisson model with an offset for the number of opportunities to exhibit the behaviours. Binary outcomes were analysed using mixed-effect Poisson regression with a log-link and robust standard errors. Continuous outcomes were analysed using a mixed-effect linear regression model. Log-transformation accommodated non-normality (microbiological findings). As a sensitivity analysis, we performed the partially adjusted analysis and fully adjusted analysis with adjustments for cluster-level baseline rates of the outcome. Baseline data were only available for mother-reported ARI and diarrhoea. Furthermore, over-dispersion in the analysis of the count outcomes was checked through sensitivity analysis, and no evidence of over-dispersion was found.

A subgroup analysis of the 32-month follow-up data, using interaction tests, was used to assess whether the intervention effect differed for children aged ≥26 months compared to those children aged <26 months (new mothers).

### Registration

The protocol was registered on 17 October 2014 with the Pan African Clinical Trial Registry in South Africa with reference PACTR201410000859336, with 32-month follow-up as an amendment to the trial.

### Trial protocol

The trial protocol for the 6-month follow-up is provided in S1 Protocol, and for the 32-month follow-up in S2 Protocol.

### Ethical approval and consent

Written and oral information was provided and informed written permission from the village head was obtained for the participation of the villages before randomisation. All mothers participating in the baseline and assessment surveys gave written informed consent. All information was read out in case of illiteracy (a written copy of the study information was left), and a thumb print was obtained in the presence of a family witness and the data collector. The study was approved by The Gambia Government/MRC Joint Ethics Committee (reference: SCC1385) and the Science, Technology, Engineering and Mathematics Ethical Review Committee at the University of Birmingham (reference: ERN 14–0574). All the information collected was kept strictly confidential, accessed only for study purposes, and analysed after personal identifiers were removed.

### Deviation from protocol

Minor changes to the protocol took place after registration, but before the start of the intervention and assessment fieldwork. These included the addition of the secondary outcome ‘diarrhoea hospitalisation in the last episode’ and the collection of cost data for child’s diarrhoea disease.

The original protocol did not explicitly plan a follow-up beyond the 6 months. As funds became available, the 32-month follow-up was added and registered as an amendment to the trial before data collection began. However, due to lack of funds, quality-assured *E*. *coli* laboratory tests on food and water and clinic data collection could not be completed (secondary outcomes). At 32 months, subgroup analysis for new mothers was conducted, given the importance of sustainability of any behaviours being practiced by new mothers. As there had been no visits by intervention or study teams since the 6-month assessment, new mothers’ adoption of practices indicated community promotion of the behaviours with new mothers who were not even pregnant at the time of the campaign.

## Results

**Fig 1:**
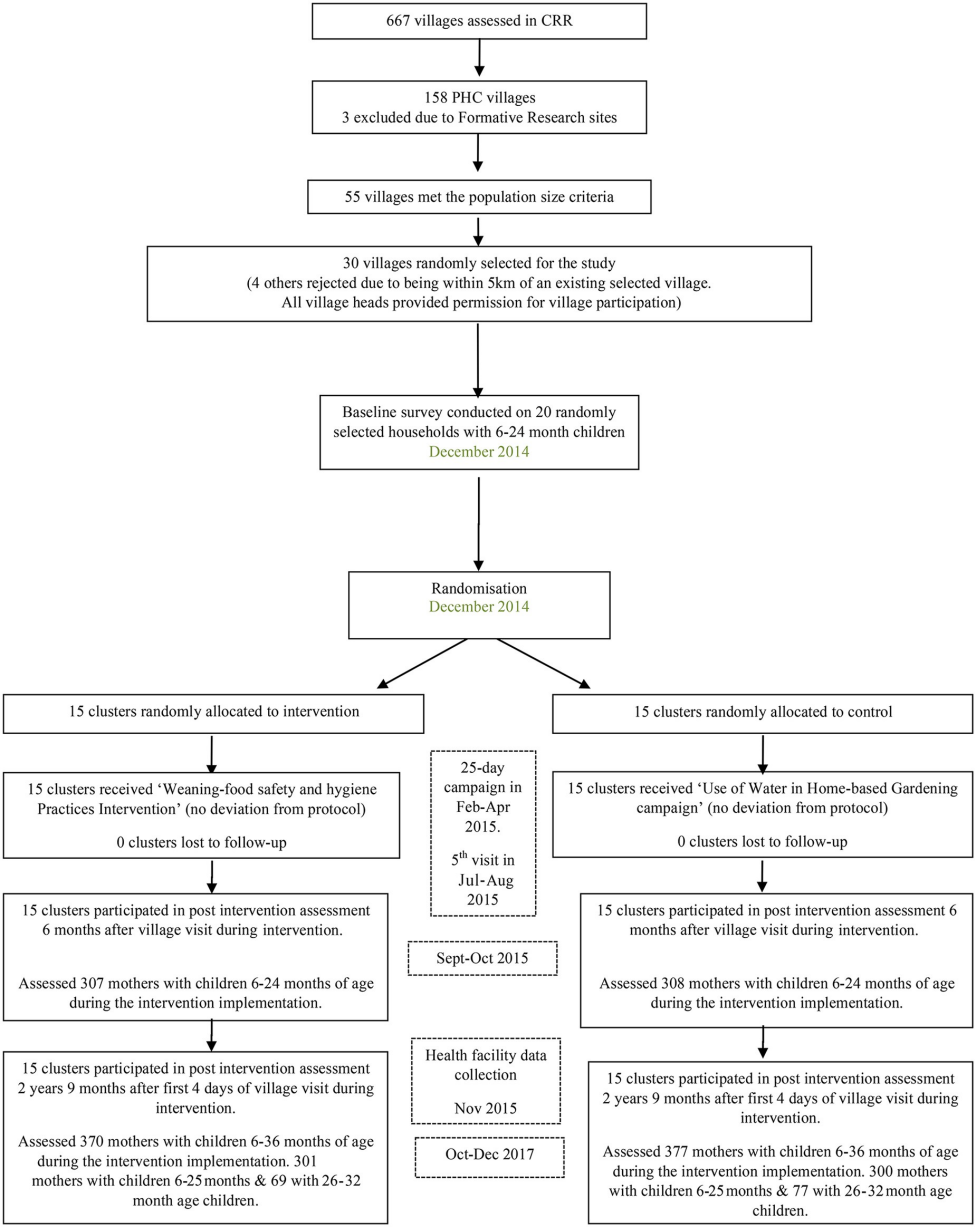
The trial flow diagram. CRR, Central River Region; PHC, Primary Health Care Programme.

Fifteen clusters were allocated to each arm (intervention and control), with 21 mothers (or a main carer) surveyed in each village at baseline [8] (total 600) and 6-month follow-up (total 615), and 22–26 mothers were surveyed per village at 32 months (total 747) (Fig 1). No village or family refused to participate, and none withdrew during observations. Mothers were the main carers in nearly all families (Table 1).

**Table 1:** Characteristics of mothers in the assessment surveys by intervention allocation.

Characteristic	6-month assessment		32-month assessment	
	Control arm *n* = 377	Intervention arm *n* = 370	Control arm *N* = 377	Intervention arm *N* = 370
Mother as the primary person responsible for complementary-food preparation and handling	396 (96%)	301 (98%)	357 (94%)	356 (98%)
Sex of child male	170 (55%)	155 (50%)	189 (53%)	211 (59%)
Age of child in months	18 [12–24]	19 [12–25]	22 [14–31]	21 [13–30]
Age of mother in years	27 [22–32]	28 [24–32]	26 [22–30]	27 [22–33]
Number of children alive for mother	3 [2–5]	3 [2–5]	3 [2–5]	4 [2–6]
Education level of mother				
None/illiterate	148 (48%)	138 (45%)	233 (62%)	206 (56%)
Other (Islamic, home, etc.)	109 (35%)	116 (38%)	91 (24%)	115 (31%)
Primary	29 (9%)	29 (9%)	32 (8%)	24 (6%)
Secondary or higher^†^	22 (7%)	24 (8%)	21 (6%)	25 (7%)
Ethnicity of mother				
Mandingo	71 (23%)	89 (29%)	60 (16%)	93 (25%)
Wolof	115 (37%)	99 (32%)	171 (45%)	121 (33%)
Fula	122 (40%)	119 (39%)	138 (37%)	151 (41%)
Occupation of mother^‡^				
Farmer	279 (91%)	285 (93%)	307 (81%)	312 (84%)
Other^§^	29 (9%)	22 (7%)	70 (19%)	58 (16%)
Number of household members	6 [5–9]	7 [5–10]	13 [9–20]	15 [10–24]
Ethnicity of husband				
Mandingo	55 (18%)	80 (26%)		
Wolof	113 (37%)	118 (38%)		
Fula	140 (45%)	109 (36%)		
Structure of house				
Mud wall, corrugated roof	134 (44%)	116 (38%)	170 (46%)	160 (43%)
Cement wall, corrugated roof	66 (21%)	84 (27%)	95 (26%)	102 (28%)
Mud wall, thatched roof	108 (35%)	107 (35%)	104 (28%)	107 (29%)
Belongings				
Land	263 (85%)	281 (92%)	318 (85%)	315 (86%)
Cattle	170 (55%)	185 (60%)	232 (62%)	253 (69%)
Goat	242 (79%)	247 (80%)	313 (83%)	345 (94%)
Mobile phone	254 (82%)	257 (84%)	332 (88%)	323 (88%)
Radio	201 (65%)	226 (74%)	249 (66%)	255 (69%)
Water tap	16 (5%)	18 (6%)	19 (5%)	41 (11%)
Refrigerator	5 (2%)	8 (3%)	8 (2%)	22 (6%)
Source of water				
Covered well	178 (58%)	161 (52%)	220 (58%)	251 (68%)
Open well	130 (42%)	146 (48%)	157 (42%)	119 (32%)
Availability of a pit latrine	286 (93%)	292 (95%)	337 (89%)	343 (93%)

Values for the individual variables are number (%) or median [IQR]. Percentages might not add to 100% due to rounding.

^†^Senior secondary or college.

^‡^All mothers were housewives, but had additional regular other work.

^§^Trading, animal husbandry, or civil servant.

The characteristics of villages and mothers were balanced between the arms at baseline [8] (S4 and S5 Tables) and at 6- and 32-month follow-up (Table 1). At baseline, in the dry season (low diarrhoea risk), approximately 24% (142/600) of mothers in both arms reported their child had at least 1 diarrhoea episode in the previous 7 days, and 10% (60/600) reported their child had ARI in the previous 7 days (see S5 Table and previous publication [8]). Similar values were found in the Multiple Indicator Cluster Survey of 2010, at 26.5% and 14.2%, respectively [13], also conducted in the dry season.

In terms of participation in the activities of the 25-day village campaign, the MaaChampion mothers competition enabled mothers to stage their behaviour change, achieving the status of pledged, sustained, and role-model mothers during the 4 visits over the 25-day village campaign (see S3 Box). At the end of the 5-month visit, of the 571 mothers of 6- to 24-month-old children in the 15 intervention villages, there were 7% (40/571) MaaFamboos (pledged mothers), 11% (63/571) MaaSawar (sustained mothers), and 51% (291/571) MaaChampions (role-model mothers). A total of 69% (394/571) of the mothers engaged at some level. All intervention villages were given the status of ‘Complementary-food Hygiene Village’ although 2 did not quite reach the 50% MaaChampion status amongst their mothers with children of complementary-food age.

For exposure to intervention in our assessed mothers, when questioned if and how the mother had learnt about complementary-food practices (with no prompting for answer options), at the 6-month follow-up 73% (223/370) of mothers in the intervention villages explicitly mentioned identifiable components of our intervention. One mother reported this in a control village.

At 6-month follow-up, the intervention led to a 4.4-fold increase in the practice of the primary outcome 5 key behaviours. The rate of recommended behaviours was 662/4,351 (incidence rate [IR] = 0.15) in the control villages versus 2,861/4,378 (IR = 0.65) in the intervention villages (adjusted IR ratio [aIRR] = 4.44, 95% CI 3.62–5.44; Table 2). There was also a substantial effect on the practice of all individual complementary-food and drinking water safety and hygiene behaviours, with aIRRs ranging from 3.27 (95% CI 2.79–3.83) for washing pots and utensils and drying them on a clean surface before food preparation to 212.20 (95% CI 52.90–852.00) for boiling the child’s drinking water (Table 2). *E*. *coli* counts on the 2 complementary-food and water samples were significantly lower in the intervention arm than in the control arm (Table 3), confirming the differences observed in the behaviour observations.

**Table 2:** The effect of the intervention on the opportunities to practise the key behaviours promoted through the intervention.

Outcome/behaviour	Number of recommended behaviours (events)		Number of opportunities for doing the behaviour		Incidence rate		Unadjusted IRR (95% CI), *p-*value	Adjusted IRR (95% CI)^†^, *p-*value	Fully adjusted IRR (95% CI)^†^, *p-*value
	C	I	C	I	C	I			
**6-month assessment**									
**Five key behaviours**^‡^	662	2,861	4,351	4,378	0.15	0.65	4.45 (3.63. 5.46) *p <* 0.001	4.44 (3.62, 5.44) *p <* 0.001	4.46 (3.63, 5.47) *p <* 0.001
**Secondary outcomes**									
Handwashing^#^ before cooking	120	394	618	613	0.19	0.64	3.32 (2.63, 4.20) *p <* 0.001	3.36 (2.67, 4.22) *p <* 0.001	3.29 (2.54, 4.26) *p <* 0.001
Handwashing^#^ during cooking if contaminated	43	469	988	992	0.04	0.47	11.17 (7.65, 16.31) *p <* 0.001	11.19 (7.65,16.37) *p <* 0.001	10.55 (7.09, 15.71) *p <* 0.001
Handwashing^#^ before feeding child	30	315	684	687	0.04	0.46	11.29 (6.81, 18.71) *p <* 0.001	11.28 (6.92,18.37) *p <* 0.001	10.25 (6.44, 16.31) *p <* 0.001
Washing pots and utensils and drying on clean surface	457	1,475	1,805	1799	0.25	0.82	3.28 (2.80, 3.85) *p <* 0.001	3.27 (2.79, 3.83) *p <* 0.001	3.31 (2.8, 3.88) *p <* 0.001
Reheating complementary food before feeding	12	209	256	288	0.05	0.73	15.48 (8.65, 27.70) *p <* 0.001	15.38 (8.59,27.53) *p <* 0.001	15.22 (8.28, 27.95) *p <* 0.001
Boiling child’s drinking water	2	428	485	498	0.00	0.86	208.70 (51.90, 838.40) *p <* 0.001	212.20 (52.90, 852.00) *p <* 0.001	188.20 (46.90, 755.80) *p <* 0.001
**32-month assessment**									
**Five key behaviours**^‡^	1,702	1,919	4,255	4,121	0.40	0.47	1.17 (1.06, 1.29) *p* = 0.002	1.17 (1.07, 1.29) *p* = 0.001	1.17 (1.07, 1.28) *p <* 0.002
**Secondary outcomes**									
Handwashing^#^ before cooking	119	181	513	504	0.23	0.36	1.55 (1.23, 1.95) *p <* 0.001	1.58 (1.26, 2.01) *p <* 0.001	1.54 (1.21, 1.96) *p =* 0.001
Handwashing^#^ during cooking if contaminated	62	120	1,012	941	0.06	0.13	2.08 (1.51, 2.86) *p <* 0.001	2.07 (1.52, 2.81) *p <* 0.001	2.01 (1.46, 2.76) *p <* 0.001
Handwashing^#^ before feeding child	32	67	728	715	0.04	0.09	2.16 (1.31, 3.56) *p =* 0.002	2.20 (1.33, 3.63) *p =* 0.002	1.99 (1.17, 3.37) *p =* 0.011
Washing pots and utensils and drying on clean surface	1,397	1,463	1,812	1,785	0.77	0.82	1.07 (0.96, 1.18) *p =* 0.217	1.07 (0.97, 1.18) *p =* 0.18	1.08 (0.98, 1.18) *p =* 0.116
Reheating complementary food before feeding	92	88	190	176	0.48	0.5	1.03 (0.77, 1.38) *p =* 0.807	1.04 (0.77, 1.39) *p =* 0.806	1.01 (0.74, 1.37) *p =* 0.949
Boiling child’s drinking water	9	167	448	472	0.02	0.35	19.60 (7.90, 48.6) *p <* 0.001	20.10 (8.30, 48.50) *p <* 0.001	20.90 (8.60, 50.40) *p <* 0.001

^†^Adjusted for cluster-level covariates used in the randomisation (location [north or south of the river] and village size).

^‡^Primary outcome. The 5 key practices were (1) handwashing with soap and water before food preparation/cooking, (2) washing of pots and utensils and drying them on a clean surface before cooking and/or serving food, (3) handwashing with soap and water during food preparation/cooking if hands became contaminated, (4) handwashing with soap and water before feeding child, and (5) reheating stored complementary-food before second feeding to the child.

^#^Handwashing with soap.

C, control clusters/villages; CI, confidence interval; I, intervention clusters/villages; IRR, incidence rate ratio.

**Table 3:** Effect of intervention on laboratory outcomes.

Sample	Median [IQR] *E*. *coli* count (CFU) per 10 g of food or 100 ml of water		Geometric mean* *E*. *coli* count (CFU) per 10 g of food or 100 ml of water		Ratio of geometric means** (95% CI), *p-*value		
	Control, *n* = 308	Intervention, *n* = 307	Control, *n* = 308	Intervention, *n* = 307	Unadjusted	Partially adjusted^†^	Fully adjusted^††^
Complementary food immediately after cooking	300 [0–3,350,000]	23 [0–2,400]	34,266	2,424	0.07 (0.00 to 1.07)	0.06 (0.00 to 0.80)	0.05 (0.00 to 0.69)
					*p =* 0.056	*p =* 0.034	*p =* 0.025
Complementary food stored before second feeding	691 [48–8,150,000]	200 [0–8,000]	49,285	5,271	0.08 (0.01 to 1.18)	0.07 (0.01 to 0.91)	0.07 (0.00 to 0.92)
					*p =* 0.066	*p =* 0.042	*p =* 0.043
Drinking water for child	30 [1–320]	1 [1–80]	25	8	0.36 (0.17 to 0.76)	0.35 (0.18 to 0.69)	0.34 (0.17 to 0.68)
					*p =* 0.007	*p =* 0.002	*p =* 0.002

*E. *coli* counts were modelled using the log10 scale. However, we have back-transformed the results to the original scale. As a result of this transformation, we are using geometric means to summarise the coliform counts in the control and intervention arm.

**The treatment effect presented here is a ratio of the geometric mean in the treatment arm compared to the geometric mean in the control arm. For example, a ratio of geometric means of 0.36 (0.17 to 0.76) suggests that the geometric mean in the intervention arm is about 36% of that under the control condition. The 95% CI of 0.17 to 0.76 suggests that the geometric mean in the intervention arm is between 17% and 76% of that under the control condition.

^†^Adjusted for cluster-level covariates used in the randomisation (location [north or south of the river] and village size).

^††**†**^Adjusted for mother’s age, mother’s education level, child’s sex, number of children in the household, and cluster-level covariates used in the randomisation (location [north or south of the river] and village size).

CFU, colony-forming units; CI, confidence interval.

The intervention led to a dramatic reduction in reported diarrhoea episodes and reported hospital admissions in the rainy season (reported diarrhoea cases 202 [66%] in the control versus 80 [26%] in the intervention villages, and reported hospitalisation cases 21[7%] versus 8 [3%], respectively; adjusted relative risk [aRR] = 0.39, 95% CI 0.32–0.48, and aRR = 0.35, 95% CI 0.19–0.66, respectively), and the risk of reported ARI (reported ARI cases 129 [42%] in the control versus 86 [28%] in the intervention villages; aRR = 0.67, 95% CI 0.53–0.86). Other outcomes (Table 4) included a higher soap availability in kitchens and in latrines in intervention villages than in control villages.

**Table 4:** Effect of the intervention on health and observed soap outcomes.

Outcome	Control *n* (%)	Intervention *n* (%)	Unadjusted RR (95% CI) *p-*value	Adjusted* RR (95% CI) *p-*value	Fully adjusted** RR (95% CI) *p-*value
**6-month assessment (control *n* = 308, intervention *n* = 307)**					
Admission for diarrhoea^‡^	21 (7%)	8 (3%)	0.38 (0.17, 0.88) *p =* 0.024	0.35 (0.19, 0.66) *p =* 0.001	0.34 (0.17,0.67) *p =* 0.001
Diarrhoea^‡‡^	202 (66%)	80 (26%)	0.40 (0.33, 0.49) *p <* 0.001	0.39 (0.32, 0.48) *p <* 0.001	0.40 (0.33, 0.49) *p <* 0.001
Admission for acute respiratory tract infection^#^	Not available				
Acute respiratory tract infection^##^	129 (42%)	86 (28%)	0.67 (0.53, 0.85) *p =* 0.001	0.67 (0.53, 0.86) *p =* 0.001	0.69 (0.54, 0.89) *p =* 0.001
Observed soap available at kitchen^†^	188 (61%)	275 (90%)	1.47 (1.29, 1.67) *p <* 0.001	1.47 (1.30, 1.66) *p <* 0.001	1.46 (1.29, 1.66) *p <* 0.001
Observed soap available at pit latrine^†^	59 (19%)	110 (36%)	1.88 (1.26, 2.79) *p =* 0.002	1.84 (1.26, 2.67) *p =* 0.001	1.84 (1.26, 2.70) *p =* 0.001
**32-month assessment (control *n* = 377, intervention *n* = 377)**					
Admission for diarrhoea^‡^	15 (6%)	5 (2%)	0.39 (0.18, 0.87) *p =* 0.021	0.38 (0.18, 0.80) *p =* 0.011	0.28 (0.12, 0.65) *p =* 0.003
Diarrhoea^‡‡^	102 (27%)	69 (19%)	0.68 (0.46, 1.02) *p =* 0.060	0.68 (0.48, 0.96) *p =* 0.027	0.68 (0.47, 0.98) *p =* 0.039
Admission for acute respiratory tract infection^#^	6 (1)	7 (2)	1.22 (0.72, 14.70) *p =* 0.173	1.17 (0.72, 14.43) *p =* 0.153	1.10 (0.46, 19.74) *p =* 0.250
Acute respiratory tract infection^##^	77 (20%)	55 (15%)	0.75 (0.48, 1.18) *p =* 0.212	0.75 (0.48, 1.17) *p =* 0.200	0.80 (0.52, 1.21) *p =* 0.284
Observed soap available at kitchen^†^	237 (64%)	270 (73%)	1.15 (0.99, 1.34) *p =* 0.060	1.17 (1.01, 1.34) *p =* 0.032	1.16 (1.01, 1.33) *p =* 0.034
Observed soap available at pit latrine^†^	171 (43%)	181 (55%)	1.27 (1.03, 1.57) *p =* 0.025	1.26 (1.04, 1.53) *p =* 0.019	1.21 (0.99, 1.48) *p =* 0.056

*Adjusted for cluster-level covariates used in the randomisation (location [north or south of the river] and village size).

**Adjusted for mother’s age, mother’s education level, child’s sex, number of children in the household, and cluster-level covariates used in the randomisation (location [north or south of the river] and village size).

^‡^Child hospital admission during the last diarrhoea episode as reported by mother.

^‡‡^Three watery stools on any day in the last 7 days as reported by mother.

^#^Child hospital admission during the last episode of cough and difficulty breathing as reported by mother.

^##^Cough and difficulty breathing on any day in the last 7 days as reported by mother.

^†^There were no explicit messages given regarding these variables during the complementary-food safety and hygiene intervention.

CI, confidence interval; RR, relative risk.

At 32 months, 66% (494/747) of mothers were new mothers, with infants born after the 5-month reminder visit (S7 and S8 Tables); a further 19% (144/747) delivered their babies after the 25-day village campaign (babies were not yet complementary-feeding age during the delivery of the intervention 25-day village campaign or the 5-month reminder visit to the village). At 32 months, in an open-ended question (without explicit mention of the programme name or activities), only 18% (67/370) of mothers in the intervention group and 6% (22/377) in the control group reported that they had heard about complementary food through programmes whose descriptions explicitly sounded like our campaign activities. There was an increase in complementary-food safety and hygiene behaviours in the control villages; for example, reheating stored complementary food was nearly never practiced during our formative research and in control villages at 6-month follow-up, but reached an IR of 48% at 32 months. This rate was similar to the 50% rate in the intervention group, thus making the IR ratio not statistically significant between the arms. The lapse of over 2 years with no programmatic activities meant that fewer mothers were practicing the behaviours in our intervention arm, and this, together with an increase in the behaviours in control villages, led to the effect sizes being reduced. There was a significant increase in the practice of the 5 key behaviours in the intervention arm (rate of recommended behaviours in the control villages was 1,702/4,255 [IR = 0.40] versus 1,919/4,121 [IR = 0.47] in the intervention villages; aIRR = 1.17, 95% CI 1.07–1.29; Table 2). There was also a higher rate of practice of all individual complementary-food and drinking-water safety and hygiene behaviours in intervention villages, with all but reheating complementary food and washing pots and utensils showing a statistically significant difference between the arms and with aIRRs ranging from 1.58 (95% CI 1.26–2.01) for washing hands before cooking to 20.10 (95% CI 8.30–48.50) for boiling the child’s drinking water (Table 2). The intervention led to a reduction in reported diarrhoea episodes and hospital admissions in the beginning of the dry season (reported diarrhoea cases of 102 [27%] in the control versus 69 [19%] in the intervention villages, and reported hospitalisation cases 15 [6%] versus 5 [2%], respectively; aRR = 0.68, 95% CI 0.48–0.96, and aRR = 0.38, 95% CI 0.18–0.80, respectively), but the risk of reported ARI, though reduced in the intervention arm, was not statistically significantly different between the arms. Soap in kitchens and latrines was also observed significantly more frequently in the intervention arm (Table 4).

For all outcomes, the precision of the estimate (i.e., the confidence interval) in the unadjusted model was changed (and mostly improved) by adjusting for cluster randomisation variables and by adjusting for additional covariates in the fully adjusted models. The sensitivity analysis adjusting for baseline reported diarrhoea and ARI was consistent with the main analysis (S6 Table).

The subgroup analysis for new mothers was consistent with the main analysis (S7 and S8 Tables). There was no evidence of interaction between intervention and child’s age (dichotomised at 26 months).

The variance component of the random effect for village for the primary composite outcome was 0.065 (SE 0.0217) at 6-month follow-up and 0.010 (SE 0.005) at 32-month follow-up.

There was no apparent harm or unintended negative or adverse events from the intervention.

The cost of material production and implementation for the intervention in 15 villages over 8 months was $16,300 (2014 US dollars), $28.53 per 6- to 24-month-old child, and $3.12 per population member at the time of the campaign (cost per head is less if new 6-month-old children for the period 6–32 months are included). This was the cost after formative research identified behaviours and motivators, and the tools were developed (the University of Birmingham is currently developing an intervention manual containing the tools). For a new cultural setting in a low- or middle-income country, an additional US$7,000–US$12,000 (as per The Gambia costs in 2018) might be required to conduct the contextualisation and adapt the tools.

## Discussion

To our knowledge we present the first trial of a complex community-level intervention of complementary-food safety and hygiene with behaviour change and health outcomes. The theory-based campaign-like and self-sustaining community intervention was implemented in rural villages of a low-income country, The Gambia. At 6 months, we found the intervention was highly effective in improving mothers’ complementary-food preparation and handling practices, reducing microbiological contamination of food and water, and increasing the availability of soap in kitchens and latrines. These findings corresponded with a reduced incidence of reported diarrhoea, diarrhoea hospitalisation, and respiratory infection. While there was no programmatic input for 26 months, the intervention behaviours were sustained and passed on to new mothers 32 months post-intervention. Reported health outcomes were still significantly improved at long-term follow-up despite the fact that the study sample size and the implementation of the intervention were not planned to detect an impact at 32 months. As expected, at 32-month follow-up, after 26 months of no contact with the programme, the rate of practice of behaviours in the intervention communities was reduced and there may have been cross-contamination in control villages for some of the behaviours. The cross-contamination means that the reduction in effect size for outcomes was more pronounced and not entirely due to a reduced rate of behaviours practiced in the intervention villages. Nevertheless, most behaviours were still practised at significantly higher rates in the intervention communities, with increased presence and use of soap in kitchens and latrines, and reduced reported diarrhoea rate and diarrhoea hospitalisation in 6- to 24-month-old children. From a public health point of view, a reduction in diarrhoea at 32 months is important given that the majority of households in sub-Saharan Africa have children <5 years old, and as a result of the need to cook food for them, diarrhoea is very common. Furthermore, the effect size was much higher at 6 months, suggesting that over the period of 32 months, the average effect size was more significant than at 32 months.

Importantly, new mothers in the intervention villages practised the key behaviours at a higher rate than those in the control villages. Qualitative data collected at 32 months (manuscript in preparation) demonstrate embedding of the programme behaviours and new mothers being taught by existing role-model mothers (MaaChampions) and older mother volunteers (MaaSupervisors), which points to the sustainability of the intervention with minimal resources.

The 6-month results are supported by clinic visit data from another study on diagnoses made at clinic visits for children <5 years old in CRR (manuscript in preparation; see S5 Box). Taal [22] retrospectively collected data on diagnoses of children visiting clinics in the first 7 months after the 25-day campaign activities, which indicated a statistically significant reduction in the incidence of cases of diarrhoea that were reported to the health facilities by our intervention versus control villagers.

Our study builds on a smaller cluster RCT of a similar but longer and more intensive intervention in Nepal, which found similar results when investigating behaviour and microbiology outcomes 1 month after the intervention [10,11]. Unpublished exploratory analysis of diarrhoea outcomes in Nepal indicated that the incidence of diarrhoea reported by mothers was also significantly reduced by more than half in the intervention villages. The small number of clusters (8) potentially limited the study generalisability and internal validity due to inflated type I errors. A systematic review of the literature revealed no comparable studies. Apart from application to industry or food outlets, HACCP analysis has been applied to household assessments [17,23–26], but only 3 small trials (all from collaborations within our group) successfully applied HACCP to developing recommended behaviour change measures for safe preparation and handling of complementary food by mothers in LMICs. Apart from the community study in Nepal, the other 2 feasibility trials were intended to demonstrate that the use of HACCP in programming and that complementary-food preparation behaviour change could reduce microbiological contamination of complementary food. The interventions were too resource intensive for programmatic purposes as they randomised mothers individually and involved teaching the behaviours to individual mothers during home visits [10,20,27]. Other trials involving complementary-food safety or hygiene were mostly quasi-experimental, were not community-based, and only involved limited aspects, such as storage interventions or the benefits of fermenting complementary food [28,29]. Otherwise, trials including aspects of food safety or hygiene primarily focussed on water, sanitation, and hygiene (WaSH) or childhood nutrition (such as handwashing before cooking or eating), without a systematic assessment of complementary-food-related hazardous behaviours before or after the interventions or an explicit focus on complementary-food safety/hygiene [30–33].

It is important to note that no matter how nutritious or deficient the complementary food is, if contaminated it is hazardous to the young child with an underdeveloped immune system. Therefore, our intervention has notable implications regardless of the nutrient composition of the child’s diet [5]. In our study, diarrhoea reduction (60%) and ARI reduction (30%) at 6-month follow-up are amongst the highest recorded from any WaSH or other community programme [30–32]. The impact on respiratory infections reflects the links between respiratory infection and handwashing [34,35]. Our findings are noteworthy since the study sample size was not initially powered to detect any difference in ARI, diarrhoea, hospitalisation reporting, or clinic visits, nor to detect any significant difference in outcomes at 32 months, especially because a dilution in effect of the intervention would be expected without further programmatic input, incentives, supervision, or re-training [22].

Our data demonstrate an apparent cross-contamination and adoption of behaviours among mothers in the control villages at 32 months. We believe this was likely to be mainly through intervention villagers promoting the ideas themselves since the regional public health office confirmed that no further promotion of complementary-food safety and hygiene had been conducted by their staff in any villages in the region (CRR) after the reminder visit at 5 months. Qualitative data at 32 months also suggests that mothers in intervention villages felt it their duty to inform mothers about complementary-food safety and hygiene practices when visiting other villages. Our nested-study data about family-food-related cooking (which showed significantly improved family-food safety behaviours in intervention villages at 32 months) further reconfirms that the behaviours were embedded into mothers’ daily practices (manuscript in preparation). This behaviour change during preparing and handling family food explains the reduced reported diarrhoea rates in older children (24–36 months), who usually start eating from the family food.

This low-cost intervention was not only acceptable to the mothers and the communities at large but was, at least in part, self-sustaining since new mothers were practicing the behaviours at 32 months. Such self-sustaining interventions over several years have rarely been investigated or demonstrated for other WaSH or nutrition intervention trials. Focus group discussions conducted at 32-month follow-up across mothers, grandmothers, and fathers (manuscript in preparation) confirmed the quantitative evidence that key behaviours were remembered by fathers and elders and were effectively communicated by villagers to new mothers, leading to further adoption of target behaviours. This was said to be driven by MaaChampions and MaaSupervisors tasked with the goal of promoting the behaviours at the fourth campaign day visit. The qualitative data indicate that community participation techniques, especially the performing arts, were important for successful intervention delivery [32]. Villagers retained fond memories of a joyous programme with songs and activities of traditional performing artists, and they treasured the achievements of MaaChampions in their family and community.

We therefore attribute the success of the intervention to the following factors: (1) the systematic focus on complementary-food safety and hygiene in programming, (2) the use of a theory-based programme utilising motivational drivers for behaviour change, (3) the application of joyous dramatic arts for portraying messages and motivational drivers, (4) whole community involvement including fathers and community leaders, and (5) peer support and education including older mothers and role-model mothers. These elements promote a change in social norms, support the mothers in their efforts to follow recommended behaviours, and substantially improve outcomes from home-visit-focussed interventions.

Key strengths of our study include a robust theoretical base to the intervention [8] (HACCP [17,18] and motivational drivers [15,16]), community involvement, and the use of culturally embedded performing arts. We provide evidence that the intervention can potentially be scaled up in similar rural settings given its low cost (S3 Box) and ease of delivery through local village and health structures (local health promotion/public health officers, traditional performing artists, and community health volunteers, as opposed to research staff). We demonstrate the ease of adapting tools previously developed in Asian programmes (S2 Table) [10,11,19], which enhances the transferability of the intervention and its wider replication across continents and cultures. As with other successful community participation programmes, monetary incentives or gifted goods were not required to secure participation [36].

A weakness inherent in such community interventions is the lack of ability to blind mothers to the intervention. However, it does not follow that lack of blinding meant that mothers or data collection teams would automatically link activities in their villages to the evaluation visits at their homes because there was no clear link made to them by the research team. Although there was no evidence of bias in carer-reported diarrhoea data, socially sensitive intervention evaluations such as those targeting hygiene are prone to reactivity bias in study participants, and it is possible that differences between groups were influenced by this bias. However, at both 6 and 32 months, the control and intervention villages were likely to have similar rates of reactivity bias as neither would expect any connection between the home visit for assessment and the intervention. The home visit at 6 months was the first long home visit observation for data collection (meaning mothers were not sensitised to study-related methods of data collection), and the data collectors were from villages not participating in the study and so were unaware of the intervention. For the 32-month visit, villagers had had no interaction with the study or intervention team for over 2 years, and over 75% of the mothers assessed had no weaning child at the time of intervention activities (new mothers). Additionally, we took exceptional care to minimise reactivity bias using established methods [19,37,38] (S2 Box) including designing the trial to reduce the exposure of the study population to the trial procedures, using random cross-sectional samples to observe mothers’ behaviours in each community (reducing the likelihood of mothers being surveyed more than once), and limiting home observations to only 2 occasions 25 months apart. To ensure there was minimal discussion about the home visits, the assessment teams stayed only 1 day in each village. To reduce mothers being affected by assessment of complementary-food safety (reactivity bias) or the staff focussing on our study outcomes (observation bias), at the 6- and 32-month assessments, the complementary-food safety and hygiene purpose of the assessment was concealed from the mothers and data collectors. This was achieved through conducting a larger assessment and embedding the complementary-food safety and hygiene assessment questions/observations within a larger evaluation of several household and village assessment components. This concealment was aided by the fact that, as in other LMICs, non-government organizations (NGOs), the government, and UN agencies, as well as research programmes like MRC Gambia, had concurrent interventions and initiatives, surveillance sites, and studies in CRR, resulting in numerous posters, visits, and surveys in villages for mothers and children [38]. Even if there were posters or signs related to complementary-food safety and hygiene, these would have been among other intervention activity posters and banners related to other government, NGO, or UN agency programmes, making it difficult for data collectors to link the survey and observations to the complementary-food intervention. Moreover, studies using direct observation to assess handwashing interventions in India [19] have shown that differential reactivity and observation are minimal if participants and assessors make no direct link between intervention and assessment.

Ideally, a formal surveillance system to collect morbidity data would be used to ensure bias in reporting was minimised, but limited resources prevented this. Child inpatient reports by mothers could not be confirmed due to poor hospital record systems in The Gambia. However, routine, unenhanced clinic data from other studies support our trial findings [22]. Therefore, we assessed internal and external validity and the overall risk of bias by comparing health outcomes (reported diarrhoea) with (1) objective microbiological outcomes at 6-month follow-up; (2) retrospectively collected clinic data [22]; (3) other environmental outcomes (soap availability in the kitchen and toilet area); (4) observed mothers’ water and complementary-food safety and hygiene behaviours; (5) mothers’ family-food safety and hygiene behaviours (at 32 months; manuscript in preparation); (6) intermediate process outcomes such as numbers of pledged mothers (MaaFamboos), sustained mothers (MaaSawars), and those achieving role-model status (MaaChampions); and (7) qualitative data at 32 months (manuscript in preparation). In our study, triangulation of all these results demonstrates the internal validity of our conclusions and indicates that the statistically significant difference we detected at 6 and 32 months was likely to be real and not due to bias or chance.

A limitation affecting the generalisability of our findings is that non-PHC villages, i.e., villages without a trained TBA and male VHWs, were not included. However, in practice, as the older mother volunteers (MaaSupervisors) were chosen by communities, they were not always the trained TBAs, and therefore, our results were not dependent on the training of TBAs and VHWs at PHC villages. Hence, we anticipate that this intervention could be readily implemented in non-PHC villages without previously trained lay people, particularly as baseline characteristics indicate that our villages were typical of The Gambia’s CRR [39]. Although this intervention is likely to be relevant to other such village settings prevalent in The Gambia, Africa, and possibly other LMICs, caution should be exercised in making conclusions about generalisability as the intervention requires testing in more diverse settings (different-sized villages, different countries, and/or urban/peri-urban settings).

Post hoc plans, and therefore a small sample size for 32-month follow-up, are less than optimal; however, we registered the 32-month follow-up before data collection and used very similar methods to the 6-month follow-up. Lack of laboratory data at 32 months is a weakness as lab tests link our health outcomes to the causal pathway of diarrhoeal diseases. However, as with the Mali, Bangladesh, and Nepal studies [10,20,27], at 6 months we demonstrate that practicing the behaviours reduced food contamination. Data on general diet or breastfeeding, which could influence children’s nutritional status, were not collected as nutritional status was not an outcome of this trial. Nonetheless, our RCT design, with balanced cluster and child/family characteristics [8], implies that dietary diversity and breastfeeding rates would likely also be balanced in the arms of the trial. Breastfeeding in CRR was prevalent, with the ‘ever breastfeeding’ rate at 98%, and 65% still breastfeeding at 6–24 months (93% breastfed at 1 year) [13].

In conclusion, we address WHO estimates that indicate a considerable global burden of FBDs, particularly affecting children <5 years of age living in LMICs. Rates of disease are highest in children aged 6–24 months, when complementary feeding is practiced. The WHO estimates of the FBD burden highlight the need for innovative interventions on improved food safety to specifically prevent infections in children of complementary-feeding age. This study describes such a strategy. This theoretically based, culturally embedded community-level complementary-food intervention was acceptable to villagers and engaged a variety of community members. Mothers and community volunteers adopted the behaviours and promoted them to new mothers over the longer follow-up period (beyond 2 years) without further programmatic input. The intervention was effective in changing mothers’ behaviour, reducing *E*. *coli* growths in complementary food and water, reported diarrhoea rates and admissions, and respiratory illness. As the differences were mainly still significant in the longer term, and both cooking of child food and diarrhoea in children are common in households in LMICs at the population level, the intervention is likely to have an important impact on child outcomes. For implementation, an annual reminder visit to the villages (as per our 5-month visit) or media activities may further improve the sustained effects of the programme. Our theory-based intervention shared significant elements with Asian interventions, indicating transferability of theory and tools across cultures. The active involvement of policy makers and public health service providers, traditional performing artists, and village authorities also contributed to a low-cost intervention programme that potentially could be successfully scaled.

There is a clear case for larger longer-term trials with health as a primary outcome, studies in different settings, and at-scale implementation studies. Such low-cost culturally embedded complementary-food safety and hygiene behaviour interventions could be a necessary component of diarrhoea prevention strategies, and the culturally embedded performing arts community interventions could be adapted for other behaviour change objectives.

## Supporting information

S1 Box Background to the country and Central River Region of The Gambia. (DOCX)

S2 Box Measures to reduce reactivity bias (RB) in mothers and observation bias (OB) in data collecting staff/data collectors. (DOCX)

S3 Box Detailed intervention description. (DOCX)

S4 Box Methods used for food microbiology. (DOCX)

S5 Box Summary of methods and findings from routine clinic data. (DOCX)

S1 CONSORT Checklist (DOCX)

S1 Protocol (PDF)

S2 Protocol (PDF)

S1 Table Details of intervention activities and duration of visits to the intervention villages. (DOCX)

S2 Table Intervention tools and their application during the intervention. (DOCX)

S3 Table Outcomes and their definitions. (DOCX)

S4 Table Baseline characteristics of the villages by intervention allocation. (DOCX)

S5 Table Characteristics of mothers in the baseline survey by intervention allocation. (DOCX)

S6 Table Effect of the intervention on outcomes with baseline data available and adjusted for baseline data. (DOCX)

S7 Table The effect of the intervention on the opportunities to practice the key behaviours promoted through the intervention among new mothers and those who were at least pregnant or had their babies during the intervention team activities. (DOCX)

S8 Table Effect of the intervention on outcomes other than observed complementary-food safety and hygiene behaviours among new mothers and those who were at least pregnant or had their babies during the intervention team activities. (DOCX)

## Abbreviations

aIRR: adjusted incidence rate ratio
ARI: acute respiratory infection
aRR: adjusted relative risk
CRR: Central River Region
FBD: food-borne disease
HACCP: Hazard Analysis Critical Control Point
IR: incidence rate
LMICs: low- and middle-income countries
PHC: Primary Health Care Programme
RCT: randomised controlled trial
TBA: traditional birth attendant
VHW: village health worker
WaSH: water, sanitation, and hygiene
