# Supplementary material for: Effects on childhood infections of promoting safe and hygienic complementary-food handling practices through a community-based programme: A cluster randomised controlled trial in a rural area of The Gambia
Source: PLoS Med. 2021 Jan 11;18(1):e1003260. doi: 10.1371/journal.pmed.1003260 (PMC7799804; doi:10.1371/journal.pmed.1003260)
Supplement: S1 Box — (DOCX) [file pmed.1003260.s001.docx]

**S1 Box: Background to the country and Central River Region of The Gambia** [8]

The cRCT was conducted in the Central River Region (CRR), one of the 7 administrative regions in The Gambia. The CRR is 48000 km^2^ in area, organised into 11 districts with a total of 659 villages, with a population of 201,506, of which 41,334 (20%) are aged <5 years [39]. All national languages and major ethnics groups are represented in CRR (Mandingka, Wolof, and Fula). CRR was selected as it has the highest incidence diarrhoea in The Gambia, particularly in children aged 6–24 months (26.5% of children aged under-5 had diarrhoea in the two weeks preceding the UNICEF Multiple Indicator Cluster Survey (MICS) in 2010, verses 17% nationally. The rates for ARI of children under-5 were 14.2% in CRR compared to 6% nationally). CRR is rural, with low literacy, and is economically the poorest region in The Gambia, its villages differ in their access to facilities such as pumped, piped or other water supply and health care. A typical village, with a head and a religious leader, has 10-60 households and a mean village size of 357(SD+59) [39].

As with other regions, UNICEF and the Ministry of Health and Social Welfare (MoH) have selected a number of villages (158 in CRR) to become Primary Health Care (PHC) villages where they have trained (for 4 weeks) a Village Health Worker (VHW) and a Traditional Birth Attendant (TBA) to provide health promotion and basic health support to the villagers.

CRR was chosen due to its high rate of childhood morbidity and mortality, and poor village infrastructure in terms of water source.
