# Supplementary material for: Effects on childhood infections of promoting safe and hygienic complementary-food handling practices through a community-based programme: A cluster randomised controlled trial in a rural area of The Gambia
Source: PLoS Med. 2021 Jan 11;18(1):e1003260. doi: 10.1371/journal.pmed.1003260 (PMC7799804; doi:10.1371/journal.pmed.1003260)
Supplement: S2 Box — (DOCX) [file pmed.1003260.s002.docx]

**S2 Box.** **Measures to reduce reactivity bias (RB) in mothers and observation bias (OB) in data collecting staff/data-collectors** [15-17].

| Bias reduced | | Measure taken |
| --- | --- | --- |
| Reactivity bias in mother (RB) | Observation bias in data-collectors (OB) |  |
| RB |  | Reduction of the study population’s exposure to the trial procedures (using random cross-sectional samples to observe mothers’ behaviours on only 3 occasions, baseline, 6- and 32-months) [15-17]. |
| RB |  | Using different research teams and methods for baseline and the assessment rounds: for baseline, male researchers surveying the mother with a short questionnaire at the door, and different teams of female data-collectors recording same questionnaire responses as a part of a longer assessment questionnaire during the 9-hour home-visit observation and survey [15-17]. |
| RB |  | Ensuring the 6- and 32-month assessment teams stayed only one day in each village [9], and thus mothers were unable to discuss data collection procedures between data collection days. |
| RB | OB | Concealment of the purpose of 6- and 32-month assessments from mothers and data-collectors by conducting a larger assessment of water and food utilisation in households and health related measures. This was facilitated as follows:   - During training and consent from mothers the survey/observations were described as a larger assessment of water and food utilisation in households and health related measures. - Not informing the data-collectors that an intervention and a trial had taken place. - Concealment of complementary-food assessment tools within a larger assessment of food and water usage observation and questionnaire tools. At 32-months additional questions and formats related to observing the child behaviour/play, ARI health economics, and details of water sources were added to the 6-month tool. - Consenting the mothers for this larger assessment and not for the complementary-food safety and hygiene trial. - Concealment from mothers and data-collectors was aided by the fact that other government, NGOs, and UN agencies and MRC Gambia have numerous concurrent nutrition/WaSH intervention programmes, studies and surveillance sites in this region resulting in numerous visits to villages for interventions and data collection. In terms of intervention material in the villages, where posters or village banners related to complementary-food safety and hygiene remained, these would have competed with other intervention activity posters and banners related to other agency programmes which are prevalent in rural LMIC communities. |
| OB |  | No contact between the study or intervention team and the villages between 6- and 32-month follow-up. This meant that at 32-months, both control and intervention villages were equally unlikely to link the 32-month assessment with the complementary-food and hygiene programme having not had any reminders or contact for over 2 years and that the families were mostly ‘new mothers’. |
