# Supplementary material for: Effects on childhood infections of promoting safe and hygienic complementary-food handling practices through a community-based programme: A cluster randomised controlled trial in a rural area of The Gambia
Source: PLoS Med. 2021 Jan 11;18(1):e1003260. doi: 10.1371/journal.pmed.1003260 (PMC7799804; doi:10.1371/journal.pmed.1003260)
Supplement: S3 Box — (DOCX) [file pmed.1003260.s003.docx]

**S3 Box. Detailed intervention description** [8,9]

1. **Intervention Summary:**

The intervention comprised four major phases:

1. A contextualisation, adaptation phase (here replaced by the formative research) where the critical control points, motivational drivers, routes of communication and cultural performing arts were defined.
2. A tool adaptation phase based on the formative research, leading to a creative brief, and adaptation of tools such as printed material, songs, stories, drama using the study material from Nepal.
3. An intervention campaign phase, delivered by health promotion/public health staff from the local health systems and local/traditional dramatic artists, consisting of 4 campaign day visits to each village on days 1, 2, 17 and 25. The campaign days focussed on mothers with children aged 6-24 months, through whole community entertainment events, meetings, home-visits and certification. Between and after the 4 visits, trained community volunteers (older mothers) and **MaaChampions** (defined below) encouraged the mothers at home. No free gifts other than a plastic sheet for drying dishes on, a small bar of soap, posters, badges and danglers were given.
4. A maintenance reminder visit at 5-months, before the diarrhoea high-risk season (in The Gambian rainy season), to maintain behaviour change.
5. **Development and design:**
   1. **Theoretical concepts used** were as follows: Hazard Analysis Critical Control Points (HACCP)(20-21) were used for the identification of corrective behaviours as they related to WHO’s ‘Five Keys to Safer Foods’ [40]. Psychological motivational drivers were applied to encourage behaviour change [15,16], while environmental cures and whole community participation facilitated behaviour change.
   2. **Formative research findings** were as follows: Critical control point (CCP) corrective behaviours and motivational drivers were defined using a combination of rapid ethnographic, observational and survey methods in pilot villages [8,9].

**Critical Control Points (CPP) and CPP Corrective measures,** prioritised after formative research [8,9] using HACCP and WHO’s ‘Five Keys to Safer Food’, were as follows:

Before food preparation:

*Handwashing with water and soap before food preparation*

*Washing of pots and utensils before food preparation and drying on a clean (and cleanable) surface*

During food preparation/cooking when hand becomes contaminated:

*Handwashing with clean water and soap when contaminated during cooking*

The way in which stored food has been stored before feeding to the child:

*Reheating of pre-made food after storage before feeding*

Before feeding the child:

*Handwashing with clean water and soap before feeding child (mother) or eating (child)*

Water ready for drinking by the child:

*Boiling and cooling of water ready for drinking by child*

**Motivational drivers for handwashing behaviour change (Evo-Eco Model)** [15,16] identified from the formative research were as follows: [8,9]

*Nurture* - the desire for a happy, thriving child

*Affiliation* - the desire to fit in with what others in a reference group are doing

*Disgust* - the desire to avoid and remove contamination

*Status* - the desire to have greater access to resources than others in the group

The findings were combined with lessons from complementary-food safety and hygiene programme in Nepal [10,11] to develop the tools and details for this low-cost and shorter public health intervention adapted to the Gambian context and delivered through existing village and health system structures.

- 1. **Design of details of the programme** were done by the research team at the University of Birmingham (including a Gambian District Public Health officer from MoH) and underwent wider consultation with expert health promotion agencies represented on a Local Scientific Advisory Committee in The Gambia (MoH, UNICEF, WHO, University of The Gambia, National Nutrition Agency (NANA), and the MRC Gambia). The content of the stories, plays, drama, songs, posters and daily routine of village campaign days were detailed and refined by the intervention team and again put to the Local Scientific Advisory Committee in The Gambia for final review.
  2. **The languages of the campaign** were the 3 local languages (Mandinka, Wolof and Fula), and the material were field-tested and piloted in one village.

1. **Intervention programme delivered during the campaign-like team visits to communities**
   1. **The intervention Team details were as follows:**

- 1 literate male and 1 illiterate female Traditional Communicator (performing artists who use traditional African drumming to communicate messages in the form of songs, dances or plays) with health promotion experience;
- 2-3 Public Health Officers (PHO) from the local Regional Public Health Team;
- 1 driver;
- 1 or more female volunteer(s) from each village who was chosen by the village elders, and often a part of the local health or village structures (usually a traditional birth attendant – (TBA)). Volunteers were called MaaSupervisors: they received 2 weeks of training and visited mothers between the campaign days.
  1. **Contact with the villages during the campaign-like intervention was as follows:**

The Intervention Team visited each village to conduct campaign activities on days 1, 2, 17 and 25 and paid a reminder visit at 5-month. The fifth visit was just before the rainy season to remind the villagers at a time when they might be so busy that they forget to practice the behaviours.

In between the intervention campaign visits, MaaSupervisors (older mother volunteers) made home-visits to (a) recruit more mothers of young children, (b) encourage engaged mothers to practice the behaviours and visit/recruit other mothers. Thus they contribute to the development of new cultural norms.

- 1. **The target audience was as follows:**

Focus was on mothers with children aged 6-24 months (the main complementary-food feeding age), but the entire community was involved in events.

The intervention was delivered to the entire village population aiming to mobilise various community members to support young mothers including other mothers, grandmothers, children and fathers.

Fathers, older women, and community leaders were actively engaged.

- 1. **The intervention thematic anchor was as follows:**

This revolved around 2 characters described in the context of an average village through story, drama and songs, (S2 Table) and demonstrated all key behaviours and motivational drivers with little reference to diarrhoea, and engendered a wish for behaviour change in village mothers as they identified with the characters’ lifestyles and behaviours.

***MaaChampion*** – a role-model mother, who practised the ‘5 key behaviours’ used in the programme (Table 1) and encouraged other mothers to do the same. She was portrayed as a good mother whose child achieved well in life and demonstrates appreciation for her/his mother’s hygiene and food safety behaviours when s/he was a child. She is also God fearing, loved by all in the community and her husband. Village mothers could achieve MaaChampion status if they managed to demonstrate their knowledge, conduct the practices and encouraged two others to do so.

***Funtu*** – a derogatory noun for a non-ideal thing, was a mother who did not practise any of the target behaviours and reaped the consequences with her family and other villagers.

These characters and other component of the programme were conveyed through activities listed in previous publications [8] (S2 Table) and in accordance with a timetable (S1 Table) [8].

- 1. **Intervention campaign activities were as follows:** (S2 Table) [8]

Detailed activities for each day are described elsewhere [8] and in S2 Table. During the 4 community visits, the HACCP corrective measures and motivational drivers were delivered using the MaaChampion theme and through performing arts (drama, songs, animations, stories, demonstrations), public pledging ceremonies, MaaChampion competition and certification or non-monetary rewards for mothers. These were presented through the following:

- Announcements in village streets
- Village wide meetings
- Neighbourhood meetings for men or women
- Home-visits.

There was also a village and MaaSupervisors certification if they achieved 50% MaaChampions amongst mothers of 6-24 month children.

- 1. **Free incentives / give-away items for mothers were limited as follows:**
- A plastic sheet (from the local market) on which they could dry dishes was given to all pledged mothers as this was a very new behaviour that was being promoted.
- Generally no soap was provided even though communities were poor. Our formative research found that soap and water for handwashing were difficult to obtain, but not real barriers, rather it was the will and motivation to obtain and use them for food preparation needs that was the challenge [8,9]. All households had some soap (e.g. for washing clothes) and research indicates giving soap does not provide sustainable change [41]. Those who achieved MaaSawar (status after pledged-mothers and before ideal mother status) received one bar of soap as a non-monetary reward.
- Posters, danglers and badges were given to women for display in the kitchen.
