# Supplementary material for: Effects on childhood infections of promoting safe and hygienic complementary-food handling practices through a community-based programme: A cluster randomised controlled trial in a rural area of The Gambia
Source: PLoS Med. 2021 Jan 11;18(1):e1003260. doi: 10.1371/journal.pmed.1003260 (PMC7799804; doi:10.1371/journal.pmed.1003260)
Supplement: S4 Box — (DOCX) [file pmed.1003260.s004.docx]

**S4 Box. Methods used for food microbiology** [20]**.**

We used an established methodology [26] for weaning food sampling and laboratory analysis.

**Sample Collection and Transportation**

**Food Sampling Procedures**:

Data collectors observing the mothers in their homes collected food samples aseptically into a sterile screwed cap container (approximately 20-30ml) while using disposable gloves and a sterile spatula (spoon). Food samples were collected after preparation before feeding the child breakfast, and again after storage before feeding again to the child as lunch (usually 3-5 hours after breakfast). The data collectors were trained to ensure proper and technically correct procedures for sample collection and to keep them in a cold box before analysis. Each day, samples were collected at 3pm from the village where data collection was being conducted. The samples were kept in a cold box for transportation to the laboratory at Bansang Hospital for analysis, or kept in a refrigerator at 4^◦^C before their processing which was within 8 hours of collection.

**Water Sampling Procedures**:

Data collectors aseptically collected household water, which was ready for the child to drink. These were collected in a sterile container with a screwed cap (20-30ml) while wearing disposable gloves. One water sample before being given to the child was collected from each household, labelled and kept in a cold box and collected with the food samples at 3pm each day. These too were transported in a cold box.

###

### Laboratory Analysis Procedure

**Food Samples Analysis**:

10gm of the sample was weighed and suspended in a sterile vial containing 90ml of sterile Maximum Recovery Diluents (1:10 ratio). The sample was then vortexed for 5 minutes to dislodge bacterial cells from the food particles. The homogenised sample was allowed to stand for 10 minutes so that heavy food particles could settle before aseptically transferring 1ml of the food supernatant to the surface of the Brilliance *E.coli*/coliform agar. The sample collected was spread by pressing gently with the spreader. The inoculated plates were incubated for 24 hour +/- 1 hour at 37°C +/- 1°C. Furthermore, all the samples with over growth due to heavy coliform contaminations were serially diluted to countable growth.

We calculated the total number of coliforms per gram by multiplying purple and pink colonies by the dilution factor. The number of presumptive Escherichia coli was obtained by multiplying the number of purple colonies by the dilution factor. The coliform count was expressed in cfu/10g unit.

#### Water Sample Analysis:

10ml of each water sample was filtered using a membrane filter (0.45 μm pore size). The filtered membrane was transferred onto an absorbent pad soaked in lauryl sulphate. The inoculated Petri dish was labelled and incubated at 37ºC for 24 hours to isolate the coliforms. We only counted yellow colonies using the horizontal grid lines. Coliform count was calculated by coliform colonies multiplied by 10 (Oxfam Delagua user manual).
