# Supplementary material for: Effects on childhood infections of promoting safe and hygienic complementary-food handling practices through a community-based programme: A cluster randomised controlled trial in a rural area of The Gambia
Source: PLoS Med. 2021 Jan 11;18(1):e1003260. doi: 10.1371/journal.pmed.1003260 (PMC7799804; doi:10.1371/journal.pmed.1003260)
Supplement: S5 Box — (DOCX) [file pmed.1003260.s005.docx]

**S5 Box. Summary of methods and findings from routine clinic data** [22]

Two months after the 6-month assessment (November-December 2015) as a part of another project, Taal retrospectively collected data from clinics in the CRR [22]. Taal investigated the diagnosis given to children visiting the CRR clinics and these included all clinics with our study villages in their catchment areas. The clinics were not previously a part of, or in any way connected with the study.

Each clinic had a register where daily patient visits were registered with basic information regarding each visit. Two trained data collectors visited each clinic and using the register and a data extraction sheet, extracted from this register in each clinic, for the period October 2014 to November 2015, data for any patient seen between the ages 0-5 years and broken down by age groups (only data for 6-24 month babies and between February 2015 (start of intervention) to November 2015 (8-10 months since the start of intervention) were used for this part of the analysis), village name, date of visit, and diagnosis.

Village data about children living in the villages during the period was collected through household census-like surveys. This data was used to estimate episodes per child years of each condition. Cluster level analysis was possible and a Poisson regression model was fitted to the data with the number of events per village as the outcome, and an offset for the number of person-years at risk. Cluster level co-variates were adjusted for.

They found that the clinic data at 6-month follow-up indicated a significant reduction of children visiting clinics for diarrhoea from the intervention villages (137 versus 292 visits/1000 child years in intervention and control villages respectively, aIRR=0.47 95%CI 0.33, 0.67, p=0.008). No significant difference for ARI was observed, though there was a reduction in the number of children attending from the intervention villages compared to control.
