# Supplementary material for: Effects on childhood infections of promoting safe and hygienic complementary-food handling practices through a community-based programme: A cluster randomised controlled trial in a rural area of The Gambia
Source: PLoS Med. 2021 Jan 11;18(1):e1003260. doi: 10.1371/journal.pmed.1003260 (PMC7799804; doi:10.1371/journal.pmed.1003260)
Supplement: S1 Protocol — (PDF) [file pmed.1003260.s007.pdf]

Promoting hygienic weaning-food handling practices through a community based programme: protocol for a cluster-randomised controlled trial in rural Gambia.

Buba Manjang[1], Karla Hemming[2], Chris Bradley[3], Jeroen Ensink[4] †, Samantha L. Burn[5], Louise J. Jackson[6], James T. Martin[7], Jama Bakari[8], Sandy Cairncross[9], Semira Manaseki-Holland[10]

[1] PhD Student, Department of Public Health, Epidemiology and Biostatistics, Institute of Applied Health Research, College of Medical and Dental Sciences, University of Birmingham, Edgbaston, Birmingham, B15 2TT, UK

[2] Senior Lecturer, Department of Public Health, Epidemiology and Biostatistics, Institute of Applied Health Research, College of Medical and Dental Sciences, University of Birmingham, Edgbaston, Birmingham, B15 2TT, UK

[3] Senior Lecturer, School of Geography, Earth and Environmental Sciences, University of Birmingham, Edgbaston, Birmingham, B15 2TT, UK

[4] *† Passed away in tragic circumstances, 29th December 2015.* Senior Lecturer, London School of Hygiene and Tropical Medicine, Keppel Street, London, WC1E 7HT, UK (Posthumous authorship)

[5] PhD Student, Harvard University, Cambridge, MA 02138, USA

[6] Research Fellow in Health Economics, Institute of Applied Health Research, University of Birmingham, Edgbaston, Birmingham, B15 2TT, UK

[7] Research Fellow in Statistics, Institute of Applied Health Research, University of Birmingham, Edgbaston, Birmingham, B15 2TT, UK

[8] Public Health Officer, Regional Health Management Office, Central River Region, The Gambia

[9] Professor, London School of Hygiene and Tropical Medicine, Keppel Street, London, WC1E 7HT, UK

[10] Clinical Senior Lecturer, Institute of Applied Health Research, College of Medical and Dental Sciences, University of Birmingham, Edgbaston, Birmingham, B15 2TT, UK

Corresponding author:
Semira Manaseki-Holland, Clinical Senior Lecturer, Institute of Applied Health Research, College of Medical and Dental Sciences, University of Birmingham, Edgbaston, Birmingham, B15 2TT, UK

s.manasekiholland@bham.ac.uk, (registered on BMJ site as manaseki@yahoo.com)

Tel: +44 121 414 4533; Fax: +44 121 414 7878

Version Notes

1.1 Published 4 April 2017

1.2 Published 14 June 2017. Changes to authorship and to *Health Economic Analysis* section (pages 11-12).

Summary: Thirty villages were the unit of randomisation for this parallel clustered controlled trial in the Gambia, each with 20 randomly selected mothers with 6-24 month old children. A community-wide intervention was delivered by a team of four who visited each village during four intensive intervention activity days in 25 days, which involved performing arts, competitions and community mobilisation. The intervention used existing health systems and village/cultural structures. The primary outcome was the observed difference between the intervention and control in the mean proportion of all five key food-related behaviours (Table 1) versus all opportunities for performing the behaviours during the observation period (hereafter called 5-behaviours). Secondary outcomes included microbiological contamination of food and water for child's consumption; the prevalence of diarrhoea and respiratory diseases; and mothers' reporting of diarrhoea admission. Two random cross-sectional samples were taken to measure baseline characteristics and outcomes: one before randomisation, and the other 6 months post-intervention.

Trial registration: The trial was registered on the 17th October 2014 with the Pan African Clinical Trial Registry in South Africa with numberPACTR201410000859336.

Keywords: cluster randomised controlled trial, diarrhoea, pneumonia, behaviour change, weaning-food, hygiene, food preparation, community intervention, performing arts, dramatic arts, motivational drives, scalability, Africa.

Background

Two small proof of concept individually randomised efficacy trials (in Mali[1] and Bangladesh[2]) used Hazard Analysis and Critical Control Points (HACCP)[3,4] to identify critical control points for improving weaning food hygiene and related mother's behaviour inculcated in a programme of individual training and follow-up of mothers. A recent community intervention conducted in Nepal[5] successfully tested such interventions as part of a community mobilisation intervention using the motivational behaviour change model[6] (based upon research in psychology that proposes ways of classifying various drivers of human behaviour) in a before-and-after cluster study. The former study was too intensive to be scalable, while the latter needs to be evaluated in a larger trial and further simplified for scaling up in size.

We describe here the design of a complex public health community intervention for our cluster-randomised control trial (cRCT). The primary objective of the trial was to investigate the effects of the complex public health community intervention that sought to improve mothers' weaning-food hygiene practices, and further to investigate the effect of the intervention on the level of microbiological contamination in food and water ready for child's consumption, the prevalence of diarrhoea and respiratory symptoms, and diarrhoea admission, as reported by the mothers.

Design, setting and population

The cRCT was conducted in the Central River Region (CRR), one of the seven administrative regions in the Gambia with a total of 659 villages, and a population of 201,506 of which 41,334 (20%) are under-5 years olds.[7] CRR was selected as it has the highest incidence of diarrhoea in the Gambia, particularly in children aged 6–24 months (26.5% of children under-5 had diarrhoea in the two weeks preceding the UNICEF Multiple Indicator Cluster Survey

(MICS) in 2010, verses 17% nationally. The rates for ARI of children under-5 were 14.2% in CRR compared to 6% nationally). As with other regions, UNICEF and the Ministry of Health and Social Welfare (MOH) have selected a number of villages (158 in CRR) to become Primary Health Care villages where they have trained (for four weeks) a Village Health Worker (VHW) and a Traditional Birth Attendant (TBA) to provide health promotion and basic health support to the villagers.[8]

Inclusion criteria for study villages were Primary Health Care villages with a population of 200–450 within CRR. Exclusions for the villages were those that were within 5km of already selected villages.

Inclusion criteria for households within the selected villages for baseline were mothers with children aged 6-24 months expecting to be resident in the village for the following six months. There were no other exclusions. Inclusion criteria for households for the evaluation of the intervention were mothers with children aged 6-24 months at the time of the four day visit of the intervention team to their village. Exclusions were the unavailability of the index child's main carer for cooking the index child's weaning-food during the evaluation visit.

Intervention

The intervention components and delivery package were theory-based (HACCP[3,4] promoted by WHO/FAO Expert Committee on Food Safety [4,9] and the motivational behaviour change model[6]), informed by local context from our formative research, and lessons/tools from community interventions in studies on hand washing in India[10] and weaning-food hygiene in Nepal.[5] Our formative research found critical control points for weaning-food hygiene behaviour change in mothers (Table 1) and that Nurture, Disgust, Affiliation, Status and Purity were the strongest motivational drives for our village mothers.[11]

For its delivery, we focussed on the use of performing arts (using culturally engrained styles of drama and songs),[12] competitions and environmental cues[13] to deliver the HACCP corrective measures and motivational drives. The details of our community weaning-food hygiene programme, designed by the research team at the University of Birmingham (including a Gambian Public Health officer from MOH), underwent wide consultation with expert health promotion agencies represented on a Local Scientific Advisory Committee in the Gambia.

Subsequently, the material was translated into the three local languages, field-tested and piloted iteratively by the intervention team in the CRR. This team, which also delivered the programme, consisted of one literate male and one illiterate female traditional communicator (cultural artists/musicians/drummers) with health promotion experience, three Public Health Officers (PHO) and an illiterate driver. The team were assisted by a female volunteer (usually a TBA) from each village, trained for two weeks, to assist during and in between the team visits. They were encouraged to find more assistant volunteers called *MaaSupervisors*.

The intervention focussed on a central role model character the “*MaaChampion*”, a mother, who practises the key behaviours used in the messages (Table 1) and encourages other mothers to do the same. Village mothers could achieve “*MaaChampion*” status if they demonstrated the same. Other components such as competitions (for mothers of children <5 years) and environmental cues were designed to embed behaviour change. The intervention was delivered to the entire population but focussed on mothers with children under-5 years in each village. It consisted of the team visiting each village on days 1, 2, 17 and 25, delivered between February and April 2015 (the dry season). A fifth visit took place after six months since we envisaged that if such a programme is to be implemented at scale, then to sustain behaviour change, villages would need a reminder visit before or early in the diarrhoea high-risk rainy season (if several months after the initial four day campaign),[14] a time when

mothers and their families are busy with farm work and may forget weaning-food hygiene behaviours. The programme's daily schedule is summarised in Tables 2.

Implementation was staggered over two months. The intervention team logged significant events, comments and overall participation of villagers/mothers in the programme that helped guide the process evaluation of the intervention implementation.

The control villages were given a day's health education campaign on water use in domestic vegetable gardening. A public health officer held community meetings explaining the campaign using a flipchart.

Outcome data collection

Evaluation of outcomes took place six months after the last of the four day village visits during the high-risk rainy season.[14] We hypothesised that if families were to retain the weaning-food practices learnt six months earlier, during this busy and high-risk period of the year, then our intervention would be successful. Independent newly recruited female fieldworkers received training for data collection. They moved in a group visiting one village per day and for each village one fieldworker was assigned to a mother who they observed for the day from approximately 6am-3pm.

An observation checklist was designed to collect data on mothers' food hygiene practices. Questionnaires were piloted and were used to collect data on the background characteristics of each family, the mother and child, data on income, health education related knowledge, the incidence of diarrhoea, ARI and hospitalisation for the index child, plus other questions that acted as diversions.

Food and water sampling

Fieldworkers collected food and water samples aseptically using the methodology described by Islam et al.[2] Samples were collected from weaning-food: i) immediately after the morning preparation before feeding the index child, ii) after storage (minimum three hours) and prior to feeding the index child. Water ready for drinking by the index child was also sampled.[2]

Outcomes and outcome measures

The primary outcome was the observed difference between the arms in the mean proportion of all five key food-related behaviours (Table 1) versus all opportunities for performing the behaviours during the observation period (6am–3pm by female fieldworkers) (hereafter called *5-behaviours*). The other outcomes were:

(1) Microbial (total coliform) growth from weaning-food after making and after storage, and from water, before being consumed by the infant.

(2) Presence of any days of diarrhoea (three watery stools in 24 hours) over the past seven days reported by the mother.

(3) Presence of any days of ARI (cough with difficulty breathing) over the past seven days reported by the mother.

(4) Reported hospital admission as the result of the last diarrhoea episode.

(5) The mean proportion of each individual food hygiene practices (including boiling water behaviour not a part of food hygiene behaviours) versus all opportunities for performing each behaviour during the observation period (6am–3pm).

Sample size

Observations during the formative research indicated that the population proportion of events in which correct behaviour was displayed (i.e. practices of heating of stored food, hand washing with soap before food preparation, during food preparation if contaminated, and before feeding the baby (measured in the first time activity occurred)) was 17/150 (11.3%). For the sample size calculation, we assumed an intra-cluster correlation coefficient (ICC) of 0.04[14] between villages and a coefficient variation of cluster size 0.22. We aimed to detect a minimum of a 25% absolute increase in behaviour in the intervention compared to the control arm. Thus with a significance level of 5% and 95% power, 15 clusters per arm, with a minimum of 12 mothers per cluster was needed.[22] Assuming loss to follow-up we intended to recruit 20 mothers within each village. In a sensitivity analysis, assuming a larger ICC of 0.1, the power (84%) remained reasonable.

Recruitment

The villages were randomly selected by a UK statistician from a list of all villages in CRR after applying the selection criteria. We provided written and oral information and received informed consent from the village heads for the participation of the villagers in the programme.

For the baseline and evaluation surveys, a list of all mothers with children between 6-24 months of age living in the village at the time was obtained from the maternal-child health register, and households were chosen randomly based on the study criteria. Mothers gave written informed consent. In case where the mother was illiterate, the information was read out (and a written copy left behind), and a thumb print obtained in the presence of a family witness and the fieldworker.

Baseline measurement

During the initial recruitment visit to the head of village (December 2014; dry season), after receiving consent, we characterised all 30 villages before randomisation. We also collected data on 20 randomly chosen mothers for the baseline survey.

Randomisation

Randomisation was conducted by a statistician in the UK: the villages were grouped and randomised within strata (north or south of the river, and by quartiles of the population size of the village) into 15 control and 15 intervention villages.

Blinding

Blinding of the implementers of the intervention programme and of families who received the intervention was not possible. However, the families were not aware of the comparative nature of the intervention with a control village.[5,10] The independent fieldworkers were newly recruited as assessors, and the weaning-food hygiene evaluation items were concealed in a package of household observation tools and questionnaires about food and water use. Furthermore, the assessors and mothers were told that the evaluation investigated domestic water and food usage. As such they were not aware of the intervention or control nature of the evaluation. The laboratory technicians were blinded, as the samples were labelled with codes.

Analysis of evaluation data

The outcome analysis will be by intention-to-treat, however, missing data will be reported and associations between outcomes explored. Mixed models will be used to adjust for clustering.[23] The outcomes are either binary (e.g. diarrhoea in the last seven days), continuous (e.g. the total coliform counts), or count (5-behaviours) and therefore logistic and linear,

mixed effect regression models will be used with a random effect for village. We will consider the comparisons to be significant at the 5% level (report 95% CIs).

For all outcomes, the primary analysis will be unadjusted for all covariates other than cluster and those covariates used in the stratified randomisation procedure (i.e. north / south of river and village size). The secondary analysis will also adjust for a set of pre-specified and clinically important covariates (age and education of mother, parity, sex of child, order of intervention implementation, and other intermediate outcomes where health outcomes are concerned).

Where available, baseline data will be inserted as a co-variate in the analysis.

Health economic analysis

If the intervention is effective in reducing the presence of infant diarrhoea and respiratory diseases, there are likely to be important economic implications. The economic analysis will compare the costs and outcomes associated with the community-based programme to promote hygienic weening-food handling practices, with the control. The primary base case analysis will adopt a societal perspective; this is important for the Gambia as out-of-pocket expenditure on healthcare is high.[15] Resource use data was collected prospectively in the trial, to estimate the costs associated with the promotion programme compared with the control. This included out-of-pocket costs, healthcare costs and the costs of the intervention. Information on unit costs or prices will be sourced to attach to each resource use item, to generate individual level cost estimates. The main economic analysis will assess cost-effectiveness based on cost per disability-adjusted life year (DALY) averted, used widely in the literature,[16;17] with a secondary analysis of cost per case of diarrhoea avoided, and cost per death due to diarrhoea averted. A decision analytic model will be used to assess the cost-effectiveness of the intervention beyond the end point of the trial. We will use a range of

cost-effectiveness thresholds, as recommended by WHO.[18] The estimates of DALY losses associated with childhood diarrhoea will be drawn from the existing literature (e.g. Salomon, et al. 2012).[17]

Strengths and weaknesses:

Strengths:

- Strongly theory based intervention
- Pragmatic intervention involving existing public health workforce in rural Gambia (low cost and easy to replicate)
- Use of culturally engaging traditional Gambian performing arts, and community mobilisation in the intervention (attractive to villagers and target mothers)

Weaknesses:

- For the trial, impossible to fully blind communities and assessors during evaluation
- Villages selected from Primary Care Villages (may pose a generalisability constraint)

References

1. Toure O, Coulibaly S, Arby A, Maiga F, Cairncross S. Piloting an intervention to improve microbiological food safety in Peri-Urban Mali. *Int J Hyg Environ Health* 2013; **216**(2): 138-45.
2. Islam MS, Mahmud ZH, Gope PS, et al. Hygiene intervention reduces contamination of weaning food in Bangladesh. *Trop Med Int Health* 2013; **18**(3): 250-8.
3. Bryan F. Hazard analysis critical control point evaluations, a guide to identifying hazards and assessing risks associated with food preparation and storage. Geneva: World Health Organization, 1992.
4. Hulebak KL, Schlosser W. Hazard Analysis and Critical Control Point (HACCP) History and Conceptual Overview. *Risk Anal*; **22**(3): 547-52.
5. Gautam O. Food hygiene intervention to improve food hygiene behaviours, and reduce food contamination in Nepal: an exploratory trial [Doctoral]. London: London School of Hygiene and Tropical Medicine, 2015.
6. Aunger R, Curtis V. Chapter 12: The Evo–Eco Approach to Behaviour Change. In: Gibson MA, Lawson DW (eds). *Applied Evolutionary Anthropology.* New York, NY: Springer; 2014, pp. 271-95.
7. Gambian Beaurau of Statistics. 2013 Population and Housing Census Preliminary Results. Serrekunda: Gambian Beaurau of Statistics, 2013.
8. Ministry of Health and Social Welfare. National Health Policy, Republic of the Gambia. "Health is Wealth". 2012-2020. Banjul: Ministry of Health and Social Welfare, 2012.
9. World Health Organization. Application of the hazard analysis critical control point (HACCP) system for the improvement of food safety. Geneva: World Health Organization, 1993.
10. Biran A, Schmidt W-P, Varadharajan KS, et al. Effect of a behaviour-change intervention on handwashing with soap in India (SuperAmma): a cluster-randomised trial. *Lancet Glob Health* 2014; **2**(3): e145-e54.
11. Manjang B. Investitgating Effectiveness of Behavioural Change Intervention in Improving Mothers Weaning Food Handling Practices: Design of a Cluster Randomized Controlled Trial in Rural Gambia. Birmingham: University of Birmingham, 2016.
12. Daykin N, Orme J, Evans D, Salmon D, McEachran M, Brain S. The impact of participation in performing arts on adolescent health and behaviour: a systematic review of the literature. *J Health Psychol* 2008; **13**(2): 251-64.
13. Abraham C. A taxonomy of behavior change techniques used in interventions. *Health Psychol* 2008; **27**(3): 379-88.
14. Brewster DR, Greenwood BM. Seasonal variation of paediatric diseases in The Gambia, west Africa. *Ann Trop Paediatr* 1993; **13**(2): 133-46.
15. Nasrin D, Wu Y, Blackwelder WC, et al. Healthcare-seeking for childhood diarrhea in developing countries: evidence from seven sites in Africa and Asia. *Am J Trop Med Hyg*. 2013; **89**(s1):3-12.
16. Edejer TT, Aikins M, Black R, Wolfson L, Hutubessy R, Evans DB. Cost effectiveness analysis of strategies for child health in developing countries. *BMJ.* 2005; **331**: 1177.
17. Salomon JA, Vos T, Hogan DR, et al. Common values in assessing health outcomes from disease and injury: disability weights measurement study for the Global Burden of Disease Study 2010. *Lancet.* 2012; **380**: 2129-43.

18. Marseille E, Larson B, Kazi DS, Kahn JG, Rosen S. Thresholds for the cost–effectiveness of interventions: alternative approaches. *Bull World Health Organ*. 2015; **93**(2): 118-24.

Table 1. Showing critical control points and corrective measures prioritised for the Gambia formative research. These became the target practices for the hygiene promotion campaign: five weaning-food hygiene and one infant drinking water hygiene practices.

**Critical Control Points**	**Corrective Measures – Behaviours the intervention aimed to improve**
Before food preparation	*Hand washing with water and soap before food preparation.*
	Washing of pots and utensils before food preparation and drying on a clean (and cleanable) surface.
Cooking	*Hand-washing with clean water and soap when contaminated during cooking.*
Food storage	*Reheating of pre-made food after storage before feeding.*
Feeding practice	*Hand-washing with clean water and soap before feeding child (mother) or eating (child).*
Water ready for drinking by child	*Boiling of water ready for drinking of child.*

Table 2. Summary of intervention activities during visits to intervention villages/cluster.

Day of village visit	Activities
1	Meeting Alkalo (village head).<br>Village Announcements about the presence of team and evening meeting.
	House-to-house visit with health volunteers and TBAs & village level drum/sing the 6 messages & invitation to pm meeting.
	**Afternoon event** at a central point for all villagers: Through drama, videos and quizzes to impart the primary purpose and the 6 messages; obtain pledges from mothers to commit to practice the promoted behaviours and aim to become *MaaChampion*.
	New community volunteers (*MaaSupervisors*) training to encourage and visit/supervise mothers of <5yr children.
2	Meeting Alkalo & announce to villagers the presence of the team.
	**House-to-house visit** with *MaaSupervisors* to check on mother's understanding & adherence to practices; promotion of mothers to *MaaFambo* or *MaaChampions*.
	**Ad-hoc women or men meeting** held separately in neighbourhoods to reinforce messages through stories and demonstrations.
3	As in day 2 plus an **afternoon event** similar to day 1.
4	As in day 3 plus an **afternoon event** similar to day 1.<br>Additionally in the **afternoon event** take Group pictures with all *MaaChampions*, *MaaSawarr* and *MaaFamboos* for the honour board.<br>During a **village-wide ceremony**, erect a weaning-food hygiene board at village entrance with drumming/campaign songs & present certificate to the Alkalo; offer villages motivational advice on sustainability by Alkalo, *MaaSupervisors* and PHOs.
5	As in day 3.
