# Supplementary material for: Effects on childhood infections of promoting safe and hygienic complementary-food handling practices through a community-based programme: A cluster randomised controlled trial in a rural area of The Gambia
Source: PLoS Med. 2021 Jan 11;18(1):e1003260. doi: 10.1371/journal.pmed.1003260 (PMC7799804; doi:10.1371/journal.pmed.1003260)
Supplement: S2 Protocol — (PDF) [file pmed.1003260.s008.pdf]

# Promoting hygienic weaning-food handling practices through a community based programme: protocol for 2 years extension follow-up for the cluster-randomised controlled trial in rural Gambia

Buba Manjang[1], Karla Hemming[2], Chris Bradley[3], Jeroen Ensink[4] †, Louise Jackson[5], James T. Martin[6], Jama Sowe[8], Sandy Cairncross[8], Semira Manaseki-Holland[9]

[1] PhD Student, Department of Public Health, Epidemiology and Biostatistics, Institute of Applied Health Research, College of Medical and Dental Sciences, University of Birmingham, Edgbaston, Birmingham, B15 2TT, UK

[2] Senior Lecturer, Department of Public Health, Epidemiology and Biostatistics, Institute of Applied Health Research, College of Medical and Dental Sciences, University of Birmingham, Edgbaston, Birmingham, B15 2TT, UK

[3] Senior Lecturer, School of Geography, Earth and Environmental Sciences, University of Birmingham, Edgbaston, Birmingham, B15 2TT, UK

[4] *† Passed away in tragic circumstances, 29th December 2015.* Senior Lecturer, London School of Hygiene and Tropical Medicine, Keppel Street, London, WC1E 7HT, UK (Posthumous authorship)

[5] Senior Lecturer in Health Economics, Department of Health Economics, Institute of Applied Health Research, College of Medical and Dental Sciences, University of Birmingham, Edgbaston, Birmingham, B15 2TT, UK

[6] Research Fellow in Statistics, Institute of Applied Health Research, University of Birmingham, Edgbaston, Birmingham, B15 2TT, UK

[7] Public Health Officer, Regional Health Management Office, Central River Region, The Gambia

[8] Professor, London School of Hygiene and Tropical Medicine, Keppel Street, London, WC1E 7HT, UK

[9] Clinical Senior Lecturer, Institute of Applied Health Research, College of Medical and Dental Sciences, University of Birmingham, Edgbaston, Birmingham, B15 2TT, UK

**<u>Corresponding author:</u>**
Semira Manaseki-Holland, Clinical Senior Lecturer, Institute of Applied Health Research, College of Medical and Dental Sciences, University of Birmingham, Edgbaston, Birmingham, B15 2TT, UK

s.manasekiholland@bham.ac.uk, (registered on BMJ site as manaseki@yahoo.com)

Tel: +44 121 414 4533; Fax: +44 121 414 7878

**Summary of 2-year follow-up protocol:** The Gambia has high rates of under-5 death from diarrhoea and pneumonia that peak during the weaning age. Community-based interventions may reduce weaning-food contamination and disease rates. A clustered randomised controlled trial was conducted in the Central River Region of the Gambia in 2012-15 to trial a community level weaning-food safety and hygiene intervention.

The initial registered protocol outlined a 6-month follow-up. A random sample of 30 villages (clusters) with a population of 200–450 was selected and after a baseline survey clusters were randomly assigned using a random number table within strata 1:1 to either – four intensive days of a community level campaign like intervention to improve weaning-food hygiene practices plus a reminder visit at 5 months, delivered through traditional Gambian performing arts and the existing public health and village structures – or to a one day community intervention campaign for use of water in domestic gardening in the control arm. The primary outcome was a composite score of five key food-related hygiene behaviours (observed), hereafter called 5-behaviours. Secondary outcomes included microbiological contamination of food and water for child's consumption; the prevalence of diarrhoea and respiratory diseases; and mothers' reporting of diarrhoea admission. Outcomes were measured at 6 months after the intervention in a cross-sectional survey of 20 randomly selected mothers with a child aged 6–24 months per village. Outcomes were compared in an intention-to-treat analysis between intervention and control group adjusted for mother's age, mother's education, sex of the index child, and number of children in the household.

Further funds were secured to follow-up to assess the ongoing effect of the intervention 2 years after the 5$^{th}$ month reminder visit. There have been no further contact with the villages after the 6-month follow-up. Methods will be similar to the 6-month follow-up. Outcomes will be measured in a cross-sectional survey of 26 randomly selected mothers with a child aged 6 to 36 months per village. The same primary and secondary outcomes measured in the

6-month follow-up will be measured at 2 years with the addition of assessing the effect of the intervention on hospitalisation from acute respiratory tract infections (ARI) and health economics analysis of ARI for the child, family-food behaviour and diarrhoea outcomes, qualitative studies. There will also be two nested cross-sectional studies examining the degree of agreement between two methods of measuring microbiological water quality and the play and care-giving behaviours related to contamination of toddler's hands outside in the yard.

**Trial registration:** The trial was registered on the 17th October 2014 with the Pan African Clinical Trial Registry in South Africa with numberPACTR201410000859336.

**Keywords:** cluster randomised controlled trial, diarrhoea, pneumonia, behaviour change, weaning-food, hygiene, food preparation, community intervention, performing arts, dramatic arts, motivational drives, scalability, Africa.

## Important Note:

**To avoid repetition and for ease of reference to the original protocol the 6-month cRCT original protocol is copied below (https://clahrcwmblog.files.wordpress.com/2017/06/protocol-manjang-et-al-promoting-hygienic-weaning-food-handling-practices-2017-ver1-2.pdf) and this is followed by the 2-year follow-up details where different from the 6-month protocol.**

**During the 6-month follow-up there were no deviations from protocol. Minor changes after registration of the trial took place, but these were before the start of intervention and evaluation fieldwork. These included: addition of the secondary outcome 'diarrhoea hospitalisation in the last episode'; and collection of cost data for child's diarrhoea disease.**

# 1. Protocol for 6-month follow-up of communities in the cRCT investigating the effect of weaning-food hygiene community intervention in rural Gambia.

## 1.1 Background

Two small proof of concept individually randomised efficacy trials (in Mali[1] and Bangladesh[2]) used Hazard Analysis and Critical Control Points (HACCP)[3,4] to identify critical control points for improving weaning-food hygiene and related mother's behaviour inculcated in a programme of individual training and follow-up of mothers. A recent community intervention conducted in Nepal[5] successfully tested such interventions as part of a community mobilisation intervention using the motivational behaviour change model[6] (based upon research in psychology that proposes ways of classifying various drivers of human behaviour) in a before-and-after cluster study. The former study was too intensive to

be scalable, while the latter needs to be evaluated in a larger trial and further simplified for scaling up in size.

We describe here the design of a complex public health community intervention for our cluster-randomised control trial (cRCT). The primary objective of the trial was to investigate the effects of the complex public health community intervention that sought to improve mothers' weaning-food hygiene practices, and further to investigate the effect of the intervention on the level of microbiological contamination in food and water ready for child's consumption, the prevalence of diarrhoea and respiratory symptoms, and diarrhoea admission, as reported by the mothers.

## 1.2 Methods:

### 1.2.1 Design, setting and population

The cRCT was conducted in the Central River Region (CRR), one of the seven administrative regions in the Gambia with a total of 659 villages, and a population of 201,506 of which 41,334 (20%) are under-5 years olds.[7] CRR was selected as it has the highest incidence of diarrhoea in the Gambia, particularly in children aged 6–24 months (26.5% of children under-5 had diarrhoea in the two weeks preceding the UNICEF Multiple Indicator Cluster Survey (MICS) in 2010, verses 17% nationally. The rates for ARI of children under-5 were 14.2% in CRR compared to 6% nationally). As with other regions, UNICEF and the Ministry of Health and Social Welfare (MOH) have selected a number of villages (158 in CRR) to become Primary Health Care villages where they have trained (for four weeks) a Village Health Worker (VHW) and a Traditional Birth Attendant (TBA) to provide health promotion and basic health support to the villagers.[8]

Inclusion criteria for study villages were Primary Health Care villages with a population of 200–450 within CRR. Exclusions for the villages were those that were within 5km of already selected villages.

Inclusion criteria for households within the selected villages for baseline were mothers with children aged 6-24 months expecting to be resident in the village for the following six months. There were no other exclusions. Inclusion criteria for households for the evaluation of the intervention were mothers with children aged 6-24 months at the time of the four day visit of the intervention team to their village. Exclusions were the unavailability of the index child's main carer for cooking the index child's weaning-food during the evaluation visit.

### 1.2.2 Intervention

The intervention components and delivery package were theory-based (HACCP[3,4] promoted by WHO/FAO Expert Committee on Food Safety [4,9] and the motivational behaviour change model[6]), informed by local context from our formative research, and lessons/tools from community interventions in studies on hand washing in India[10] and weaning-food hygiene in Nepal.[5] Our formative research found critical control points for weaning-food hygiene behaviour change in mothers (Table 1) and that Nurture, Disgust, Affiliation, Status and Purity were the strongest motivational drives for our village mothers.[11]

For its delivery, we focussed on the use of performing arts (using culturally engrained styles of drama and songs),[12] competitions and environmental cues[13] to deliver the HACCP corrective measures and motivational drives. The details of our community weaning-food hygiene programme, designed by the research team at the University of Birmingham (including a Gambian Public Health officer from MOH), underwent wide consultation with expert health promotion agencies represented on a Local Scientific Advisory Committee in the Gambia.

Subsequently, the material was translated into the three local languages, field-tested and piloted iteratively by the intervention team in the CRR. This team, which also delivered the programme, consisted of one literate male and one illiterate female traditional communicator (cultural artists/musicians/drummers) with health promotion experience, three Public Health Officers (PHO) and an illiterate driver. The team were assisted by a female volunteer (usually a TBA) from each village, trained for two weeks, to assist during and in between the team visits. They were encouraged to find more assistant volunteers called *MaaSupervisors*.

The intervention focussed on a central role model character the "*MaaChampion*", a mother, who practises the key behaviours used in the messages (Table 1) and encourages other mothers to do the same. Village mothers could achieve "*MaaChampion*" status if they demonstrated the same. Other components such as competitions (for mothers of children <5 years) and environmental cues were designed to embed behaviour change. The intervention was delivered to the entire population but focussed on mothers with children under-5 years in each village. It consisted of the team visiting each village on days 1, 2, 17 and 25, delivered between February and April 2015 (the dry season). A fifth visit took place after six months since we envisaged that if such a programme is to be implemented at scale, then to sustain behaviour change, villages would need a reminder visit before or early in the diarrhoea high-risk rainy season (if several months after the initial four day campaign),[14] a time when mothers and their families are busy with farm work and may forget weaning-food hygiene behaviours. The programme's daily schedule is summarised in Tables 2.

Implementation was staggered over two months. The intervention team logged significant events, comments and overall participation of villagers/mothers in the programme that helped guide the process evaluation of the intervention implementation.

The control villages were given a day's health education campaign on water use in domestic vegetable gardening. A public health officer held community meetings explaining the campaign using a flipchart.

### 1.2.3 Outcome data collection

Evaluation of outcomes took place six months after the last of the four day village visits during the high-risk rainy season.[14] We hypothesised that if families were to retain the weaning-food practices learnt six months earlier, during this busy and high-risk period of the year, then our intervention would be successful. Independent newly recruited female data collectors received training for data collection. They moved in a group visiting one village per day and for each village one data collector was assigned to a mother who they observed for the day from approximately 6am-3pm.

An observation checklist was designed to collect data on mothers' food hygiene practices. As the baby food is made at the same time as family breakfast, the practices were those for preparing morning's food. Questionnaires were piloted and were used to collect data on the background characteristics of each family, the mother and child, data on income, health education related knowledge, the incidence of diarrhoea, ARI and hospitalisation for the index child, plus other questions that acted as diversions.

### 1.2.4 Food and water sampling

Data collectors collected food and water samples aseptically using the methodology described by Islam et al.[2] Samples were collected from weaning-food: i) immediately after the morning preparation before feeding the index child, ii) after storage (minimum three hours) and prior to feeding the index child. Water ready for drinking by the index child was also sampled.[2]

### 1.2.5 Outcomes and outcome measures

The primary outcome was the observed difference between the arms in the mean proportion of all five key food-related behaviours (Table 1) versus all opportunities for performing the behaviours during the observation period (6am–3pm by female data collectors) (hereafter called *5-behaviours*). The other outcomes were:

(1) Microbial (total coliform) growth from weaning-food after making and after storage, and from water, before being consumed by the infant.

(2) Presence of any days of diarrhoea (three watery stools in 24 hours) over the past seven days reported by the mother.

(3) Presence of any days of ARI (cough with difficulty breathing) over the past seven days reported by the mother.

(4) Reported hospital admission as the result of the last diarrhoea episode.

(5) The mean proportion of each individual food hygiene practices (including boiling water behaviour not a part of food hygiene behaviours) versus all opportunities for performing each behaviour during the observation period (6am–3pm).

### 1.2.6 Sample size

Observations during the formative research indicated that the population proportion of events in which correct behaviour was displayed (i.e. practices of heating of stored food, hand washing with soap before food preparation, during food preparation if contaminated, and before feeding the baby (measured in the first time activity occurred)) was 17/150 (11.3%). For the sample size calculation, we assumed an intra-cluster correlation coefficient (ICC) of 0.04[14] between villages and a coefficient variation of cluster size 0.22. We aimed to detect a minimum of a 25% absolute increase in behaviour in the intervention compared to the control arm. Thus with a significance level of 5% and 95% power, 15 clusters per arm, with a

minimum of 12 mothers per cluster was needed.[22] Assuming loss to follow-up we intended to recruit 20 mothers within each village. In a sensitivity analysis, assuming a larger ICC of 0.1, the power (84%) remained reasonable.

### 1.2.7 Recruitment

The villages were randomly selected by a UK statistician from a list of all villages in CRR after applying the selection criteria. We provided written and oral information and received informed consent from the village heads for the participation of the villagers in the programme.

For the baseline and evaluation surveys, a list of all mothers with children between 6-24 months of age living in the village at the time was obtained from the maternal-child health register, and households were chosen randomly based on the study criteria. Mothers gave written informed consent. In case where the mother was illiterate, the information was read out (and a written copy left behind), and a thumb print obtained in the presence of a family witness and the data collector.

### 1.2.8 Baseline measurement

During the initial recruitment visit to the head of village (December 2014; dry season), after receiving consent, we characterised all 30 villages before randomisation. We also collected data on 20 randomly chosen mothers for the baseline survey.

### 1.2.9 Randomisation

Randomisation was conducted by a statistician in the UK: the villages were grouped and randomised within strata (north or south of the river, and by quartiles of the population size of the village) into 15 control and 15 intervention villages.

### 1.2.10 Blinding

Blinding of the implementers of the intervention programme and of families who received the intervention was not possible. However, the families were not aware of the comparative nature of the intervention with a control village.[5,10] The independent data collectors at evaluation were newly recruited as assessors, and the weaning-food hygiene evaluation items were concealed in a package of household observation tools and questionnaires about food and water use. Furthermore, the assessors and mothers were told that the evaluation investigated domestic water and food usage. As such they were not aware of the intervention or control nature of the evaluation. The laboratory technicians were blinded, as the samples were labelled with codes.

### 1.2.11 Analysis of evaluation data

The outcome analysis will be by intention-to-treat, however, missing data will be reported and associations between outcomes explored. Mixed models will be used to adjust for clustering.[23] The outcomes are either binary (e.g. diarrhoea in the last seven days), continuous (e.g. the total coliform counts), or count (5-behaviours). The primary outcome (5-behaviours) will be analysed using a mixed effect Poisson regression model, with the number of completed behaviours as the outcome, and an offset for the number of opportunities to exhibit the behaviour. For all secondary outcomes, we will use a mixed effect linear regression model for continuous outcomes, and a Poisson mixed effect model with a log link for binary outcomes. All models will contain robust standard errors to allow for the misspecification of the variance. Data will be transformed, where necessary, if the transformation will improve the model. We will consider the comparisons to be significant at the 5% level (report 95% CIs).

For all outcomes, the primary analysis will be unadjusted for all covariates other than cluster and those covariates used in the stratified randomisation procedure (i.e. north / south of river

and village size). The secondary analysis will also adjust for a set of pre-specified and clinically important covariates (age and education of mother, parity, sex of child, order of intervention implementation, and other intermediate outcomes where health outcomes are concerned).

Where available, baseline data will be inserted as a co-variate in the analysis. All analyses will be conducted using Stata 15.

### 1.2.12 Deviation from original analysis plan

Rather than using logistic models, a Poisson model will be used for the (primary) count outcome, and a Poisson model with a log link for the (secondary) binary outcomes. This allows the results to be presented as an incidence rate ratio and as a relative risk, rather than an odds ratio – which will allow the results to be more easily interpreted.

### 1.2.13 Health economic analysis

If the intervention is effective in reducing the presence of infant diarrhoea and respiratory diseases, there are likely to be important economic implications. The economic analysis will compare the costs and outcomes associated with the community-based programme to promote hygienic weening-food handling practices, with the control. The primary base case analysis will adopt a societal perspective; this is important for the Gambia as out-of-pocket expenditure on healthcare is high. Resource use data was collected prospectively in the trial, to estimate the costs associated with the promotion programme compared with the control. This included out-of-pocket costs, healthcare costs and the costs of the intervention. Information on unit costs or prices will be sourced to attach to each resource use item, to generate individual level cost estimates. The main economic analysis will assess cost-effectiveness based on cost per disability-adjusted life year (DALY) averted, used widely in the literature, with a secondary analysis of cost per case of diarrhoea avoided, and cost per

death due to diarrhoea averted. A decision analytic model will be used to assess the cost-effectiveness of the intervention beyond the end point of the trial. We will use a range of cost-effectiveness thresholds, as recommended by WHO. The estimates of DALY losses associated with childhood diarrhoea will be drawn from the existing.

**Table 1.** Showing critical control points and corrective measures prioritised for the Gambia formative research. These became the target practices for the hygiene promotion campaign: five weaning-food hygiene and one infant drinking water hygiene practices.

| **Critical Control Points** | **Corrective Measures – Behaviours the intervention aimed to improve** |
|---|---|
| Before food preparation | *Hand washing with water and soap before food preparation.* |
| | *Washing of pots and utensils before food preparation and drying on a clean (and cleanable) surface.* |
| Cooking | *Hand-washing with clean water and soap when contaminated during cooking.* |
| Food storage | *Reheating of pre-made food after storage before feeding.* |
| Feeding practice | *Hand-washing with clean water and soap before feeding child (mother) or eating (child).* |
| Water ready for drinking by child | *Boiling of water ready for drinking of child.* |

**Table 2.** Summary of intervention activities during visits to intervention villages/cluster.

| Day of village visit | Activities |
|---|---|
| 1 | Meeting Alkalo (village head).<br>Village Announcements about the presence of team and evening meeting. |
| | House-to-house visit with health volunteers and TBAs & village level drum/sing the 6 messages & invitation to pm meeting. |
| | **Afternoon event** at a central point for all villagers: Through drama, videos and quizzes to impart the primary purpose and the 6 messages; obtain pledges from mothers to commit to practice the promoted behaviours and aim to become *MaaChampion*. |
| | New community volunteers (*MaaSupervisors*) training to encourage and visit/supervise mothers of <5yr children. |
| 2 | Meeting Alkalo & announce to villagers the presence of the team. |
| | **House-to-house visit** with *MaaSupervisors* to check on mother's understanding & adherence to practices; promotion of mothers to *MaaFambo* or *MaaChampions*. |
| | **Ad-hoc women or men meeting** held separately in neighbourhoods to reinforce messages through stories and demonstrations. |
| 3 | As in day 2 plus an **afternoon event** similar to day 1. |
| 4 | As in day 3.<br>Additionally in the **afternoon event** take Group pictures with all *MaaChampions*, *MaaSawarr* and *MaaFamboos* for the honour board.<br>During a **village-wide ceremony**, erect a weaning-food hygiene board at village entrance with drumming/campaign songs & present certificate to the Alkalo; offer villages motivational advice on sustainability by Alkalo, *MaaSupervisors* and PHOs. |
| 5 | As in day 3. Plus motivate community volunteers and MaaChampions to continue their work beyond the project team visits. |

# 2. Protocol for the second evaluation of clusters at 2-year follow-up investigating the effect of weaning-food hygiene community intervention in rural Gambia

**Introduction**

The objective is to ascertain any residual effect from the intervention for all the outcome measures at 30-months follow-up since the 4 days campaign activities and 2-year follow-up since the reminder 5th-month visit was carried out. (Intervention was village visits on days 1,2,17,25 and volunteer home visits in between, constituting the main intervention hereafter called the 4-day village campaign visits, plus a reminder visit at the 5th-month after these initial 25 days).

For the main cRCT outcome measures, the procedure for evaluation at 6-months will be repeated by a new recruit of 26 female data collectors who will be trained as with the pervious group of data collectors at 6month. The same tools will be used with minor changes for 2 years as appropriate. However, some additional secondary research questions and sub-studies with respect to the index child and adults have been added as described below. Two nested studies took place at the same time also which helped to mask the data collection for the trial outcomes.

## 2.1 Quantitative studies

### 2.1.1 Acute respiratory infection (ARI) hospitalisation data and family costs

**2.1.1.1 Secondary research question:** Is there reduced hospitalisation from ARI (during the last episode) in the intervention compared to control arms?

**2.1.1.2 Outcomes**: Hospitalisation during the last episode of ARI (cough with difficulty breathing) as reported by the mother. ARI hospitalisation has been added because of the

finding of a 30% reduction in self-reported ARI at 6 months post-intervention plus a statistically significant reduction in hospitalisation for diarrhoea. If the hygiene practices are continued at high rates, hospitalisation for ARI may also be reduced at 2 years.

**Methods:** One question is added about the hospitalisation of the child during its last ARI episode defined in the same way as in the 6-month follow-up.

#### 2.1.1.3 Additional ARI health economic analysis of 2 years data

If the intervention is effective in reducing the presence of infant respiratory diseases as was shown at 6 months, there are likely to be important economic implications.

**Methods:** The methods and questions in the structured questionnaire tool will be similar to the diarrhoea health economic analysis component described in the 6-month analysis. Only the questionnaire has had components added about child's ARI costs (cost to the family) and these questions are similar to those for diarrhoea that were tested and had received ethical approval.

### 2.1.2 Adult/family-food handling/preparation hygiene practices, coliform growth of family-food samples, diarrhoea in mother as secondary outcome

**2.1.2.1 Secondary research question:** Has the weaning-food hygiene community intervention improved safety and hygienic preparation and handling of family-food by the mother and the rate of diarrhoea of the mother?

#### 2.1.2.2 Family-food Outcomes (similar to weaning-food outcomes):

(1) The observed mean proportion of **all five key food-related behaviours** as a fraction of all opportunities for performing the behaviours during the observation period (6am–3pm by

female data collectors) (Table 1; practices 1-5 and not including boiling water since water is often boiled for tea anyway; hereafter called *5-behaviours*).

(2) The observed mean proportion of **each of the individual five** key food-related behaviours (Table 1; practices 1-5 and including boiling water since water is often boiled for tea anyway) as a fraction of all opportunities for performing each behaviour during the observation period (6am–3pm by female data collectors) (hereafter called *5-behaviours*).

(3) Any days of diarrhoea (three watery stools in 24 hours) over the past seven days reported by the mother for the mother herself.

Our 6-month follow-up evaluation design which is to be repeated at 2 years included observing the cooking of the family breakfast and lunch and taking samples of the family-food at breakfast and at lunch (as well as the weaning-food samples and water). This was used to mask the data collectors observing the mothers' weaning-food hygiene practices and masking the mothers since our intervention messages were only about weaning-food and family food data were not monitored rigorously nor analysed.

In the 2-year follow-up this exploratory data will be quality checked and analysis has been added to our outcomes, since preliminary data from our pilot village indicate that after 2 years post intervention, the villagers apply the behaviours and for some of the families this has influenced the way mothers prepare the food for the whole family. Given the likelihood of an improvement in family-food hygiene practices at 2-year follow-up, we intend to analyse the adult/family-food related behaviours during cooking and food handling observations of family meals at 2-year follow-up.

**Methods:** Only the question about mother's diarrhoea is added in the first page of the questionnaire for the 2 years tools since the consent form, and observations, from the 6-month follow-up already contained family-food components.

**2.1.3 The population sample of mothers per village for 2 years evaluation data (including additional variables in 2.1.1 and 2.1.2)**

We aim to select mothers who are currently weaning or have children who may have a combination of weaning and family food (E.g. 24-36month old children). As such some will be those who would not have been prgrnant or had an infant at the time of the 4-days campaign visits or the 5th-month reminder.

For sampling frame, as with the 6-month follow-up the baseline list of children in the villages will be updated using the maternal-child health register and village health worker's list of children in each village. From these there will be 26 mothers chosen from each of the control and intervention villages. Sample size will be 23-26 mothers per cluster (as suppose to the target of 20 at the 6-month follow-up) because as indicated in the 6-month sample size calculation a minimum of 12 mothers per cluster of weaning children is needed (see above sample size for 6-month follow-up) but at 2 years we have 2 groups of mothers: new mothers and those who are mothers at the time of the 4-days campaign activities. (New mothers at 2-year follow-up are those with children 6-25month who will be eating weaning-food. Children aged 26-36 months will still, for the most part, be eating weaning-food at 2-year follow-up plus some of the family-food. Their mothers would have been pregnant during the 4-days campaign visits of the intervention in December to February 2014 and ready to wean at the 5th-month reminder visit. This population are the candidates most likely to *remember and practice* the hygienic behaviours while still *making* weaning-food during the 2-year assessment.) Therefore, we aim to have approximately 12 of each sub-group of mothers recruited. All individual mothers or main carers of children 6 to 36 months living in the village for a minimum of 2 years will be eligible for selection but mothers who are not available for cooking the child's weaning-food during the 2-year evaluation visit will be

excluded. (All mothers were available for the 6-month evaluation survey so we envisage very few or no mothers will be excluded).

To capture all these sub-groups of mothers, the selection of the mothers will be stratified as follows:

1. All mothers of children age 18 to 24 months will be chosen.
2. The rest of the mothers will be chosen according to the following formula:

   $$x = \frac{(26 - \text{all mothers with 18 to 24 month old children})}{2}$$

3. Then, $x$ number of mothers with children aged 6-18 months and $x$ number of mothers with children aged 24-36 months will be randomly chosen from the list.

For sub-group analysis, ‘New mothers’ will be classified as those with children 6-25m since these mothers would not have had a child at before the 5$^{th}$ village reminder visit and the 6-month evaluation and so as mothers were not a part of the MaaChampian program when any part of the intervention was taking place in the villages.

### 2.1.4 Blinding/masking at assessment

Further to the masking during assessment at 6-months, the same methods were employed to mask the assessment team and mothers to the 32-month data collection purpose as follows:

- Reduction of the study population’s exposure to the trial procedures (using random cross-sectional samples to observe mother’s behaviours on only 3 occasions, baseline, 6 and 32 months).[21]

- Using different research teams and methods for baseline and each of the evaluation rounds: For baseline, male researchers surveying the mother with a short questionnaire at the door, and two different teams of female data-collectors.[21]

- Ensuring the baseline, 6- and 32-month evaluation teams stayed only one day in each village,[9] and thus mothers were unable to discuss assessor procedures between data collection days.

- Concealment of the purpose of 6- and 32-month evaluations from mothers and data-collectors by conducting a larger evaluation of water and food utilisation in households and health related measures. This was facilitated as follows:

    - During training and consent from mothers describing the survey/observations as a larger evaluation of water and food utilisation in households and health related measures.
    - Not informing the data-collectors that an intervention and a trial has taken place.
    - Concealment of weaning-food evaluation assessments tools within a larger assessment of food and water usage observation and questionnaire tools. At 32-months additional questions and formats related to observing the child behaviour/play, ARI health economics, and details of water sources were added to the 6-month tool.
    - Consenting the mothers for this larger evaluation and not for the weaning-food safety and hygiene trial
    - Concealment from mothers and data-collectors aided by the fact that other government, NGO, and UN agencies and MRC Gambia have numerous concurrent nutrition/WaSH intervention programmes, studies and surveillance sites in this region resulting in numerous visits to villages for interventions and data collection. In terms of intervention material in the villages, if some posters or village banners related to weaning-food safety

and hygiene remained, these would have been simultaneous with other intervention activity posters and banners related to other agency programmes as is prevalent in LMIC rural communities.

- No contact between the study or intervention team and the villages between 6- and 32-month follow-up. This meant that at 32-months, both control and intervention villages were equally unlikely to link the 32-month evaluation with the weaning-food and hygiene programme having not had any reminders or contact for over 2 years and that the families were mostly ‘new mothers’.

#### 2.1.5 Statistical analysis for 2 years evaluation data (including additional variables in 2.1.1 and 2.1.2)

The statistical analysis plan for the analysis of the 2-year follow-up evaluation data will follow the same analysis plan described earlier for the 6-month follow-up evaluation data.

Sub-group analysis will be performed to establish the effect on the ‘new mothers’.

All analyses will be conducted using Stata 15.

## 2.2 Qualitative Studies:

**2.2.1 Background**: Often community participation programmes are not assessed for the success of individual elements. We hope that this assessment will guide us to improve and expand the successful elements of the programme.

**2.2.2 Objectives**: The objectives of this qualitative process assessment are to investigate the elements of the intervention the villagers felt were most effective and attractive/memorable to them (encouraging them to attend the village meetings), and what messages they still retain.

**2.2.3 Methods**: After the new independent quantitative data collector team have finished visiting the villages for evaluation, focus group discussions (FGD) will be conducted with mothers, grandmothers, and fathers in 3 or 4 intervention villages, One FGD will be held with Alkalos and one will be held with TBAs brought together from 6-10 intervention villages (total 11-14 FGD depending on saturation of themes emerging from mothers/grandmothers/father's FGDs).

FGDs will be conducted by 2 trained researchers not involved in the intervention or 6-month and 2-year follow-up. They will be conducted in the local languages and facilitated by a researcher who speaks these languages fluently. These discussions will be audio recorded and later transcribed/translated into English. Anonymised clips of audio recordings will then be sent to an independent language interpreter to validate translation. Translated FGD data will be analysed thematically by independent researchers. NVIVO software will be used to assist with data analysis.

## 1.2.15 References

1. Toure O, Coulibaly S, Arby A, Maiga F, Cairncross S. Piloting an intervention to improve microbiological food safety in Peri-Urban Mali. *Int J Hyg Environ Health* 2013; **216**(2): 138-45.
2. Islam MS, Mahmud ZH, Gope PS, et al. Hygiene intervention reduces contamination of weaning-food in Bangladesh. *Trop Med Int Health* 2013; **18**(3): 250-8.
3. Bryan F. Hazard analysis critical control point evaluations, a guide to identifying hazards and assessing risks associated with food preparation and storage. Geneva: World Health Organization, 1992.
4. Hulebak KL, Schlosser W. Hazard Analysis and Critical Control Point (HACCP) History and Conceptual Overview. *Risk Anal*; **22**(3): 547-52.
5. Gautam O. Food hygiene intervention to improve food hygiene behaviours, and reduce food contamination in Nepal: an exploratory trial [Doctoral]. London: London School of Hygiene and Tropical Medicine, 2015.

6. Aunger R, Curtis V. Chapter 12: The Evo–Eco Approach to Behaviour Change. In: Gibson MA, Lawson DW (eds). *Applied Evolutionary Anthropology*. New York, NY: Springer; 2014, pp. 271-95.
7. Gambian Beaurau of Statistics. 2013 Population and Housing Census Preliminary Results. Serrekunda: Gambian Beaurau of Statistics, 2013.
8. Ministry of Health and Social Welfare. National Health Policy, Republic of the Gambia. "Health is Wealth". 2012-2020. Banjul: Ministry of Health and Social Welfare, 2012.
9. World Health Organization. Application of the hazard analysis critical control point (HACCP) system for the improvement of food safety. Geneva: World Health Organization, 1993.
10. Biran A, Schmidt W-P, Varadharajan KS, et al. Effect of a behaviour-change intervention on handwashing with soap in India (SuperAmma): a cluster-randomised trial. *Lancet Glob Health* 2014; **2**(3): e145-e54.
11. Manjang B. Investitgating Effectiveness of Behavioural Change Intervention in Improving Mothers Weaning-food Handling Practices: Design of a Cluster Randomized Controlled Trial in Rural Gambia. Birmingham: University of Birmingham, 2016.
12. Daykin N, Orme J, Evans D, Salmon D, McEachran M, Brain S. The impact of participation in performing arts on adolescent health and behaviour: a systematic review of the literature. *J Health Psychol* 2008; **13**(2): 251-64.
13. Abraham C. A taxonomy of behavior change techniques used in interventions. *Health Psychol* 2008; **27**(3): 379-88.
14. Brewster DR, Greenwood BM. Seasonal variation of paediatric diseases in The Gambia, west Africa. *Ann Trop Paediatr* 1993; **13**(2): 133-46.
15. Hounton S, Newlands D. Applying the Net-Benefit Framework for Analyzing and Presenting Cost-Effectiveness Analysis of a Maternal and Newborn Health Intervention. *PLoS One* 2012; **7**(7).
16. Marseille E. Thresholds for the cost-effectiveness of interventions: alternative approaches. *Bull World Health Organ* 2015; **93**(2): 118-25.
17. Jamison DT. Breman JG, Measham AR, Alleyne G, Claeson M, Evans DB, et al. Disease control priorities in developing countries, 2$^{nd}$ edition [electronic resource]. Washington, DC: The International Bank for Reconstruction and Development / The World Bank; New York, NY: Oxford University Press, 2006.

August 2017
