# Supplementary material for: Effects on childhood infections of promoting safe and hygienic complementary-food handling practices through a community-based programme: A cluster randomised controlled trial in a rural area of The Gambia
Source: PLoS Med. 2021 Jan 11;18(1):e1003260. doi: 10.1371/journal.pmed.1003260 (PMC7799804; doi:10.1371/journal.pmed.1003260)
Supplement: S1 Table — (DOCX) [file pmed.1003260.s009.docx]

**S1 Table. Details of intervention activities and duration of visits to the intervention villages** (cited from our former publication [8]).

The idea of 4 visits on days 1, 2, 17, 25 was adapted from the Indian SuperAmma study [19]. However, the details of events were adapted mainly from the Complementary-food safety and hygiene Nepal study [10,11], itself drawing on aspects from the SuperAmma India study (see footnote to S2 Table for source of adapted tools) [8].

| **Event** | | **Activity** | **Where** | **Time** | **Purpose** |
| --- | --- | --- | --- | --- | --- |
| Day 1 | Meeting the Alkalo (village head) | ► TCs play a song in praise of the Alkalo.  ► The team greet the Alkalo.  ► Explain purpose/project.  ► Meet VHW and TBA and MaaSupervisor. | Alkalo’s residence | 20 min | ► Alkalo is the entry point to the village; must receive a visit before start of work.  ► Alkalo and wife have social status & their support motivates mothers. |
|  | Announcement to the villagers | ► TCs invite villagers to afternoon meeting by drumming and campaign song with the use of a loud speaker. | Within whole village | 2 hours | ► Create alert.  ► Mobilise the community.  ► Assist memorisation by repetition of song and behaviours. |
|  | House-to-house visit with MaaSupervisors | ► House-to-house visit (invite household members to afternoon meeting) with TBA and VHW. | Residence of every household especially with young children | 3 hours | ► Social mobilisation to involve the whole community. |
|  | Record a short video | ► Video the Alkalo and wife handwashing and reheating complementary-food to show at the village meeting later. | Alkalo’s residence | 15 min | ► Alkalo and wife have social status and their support motivates mothers engender a social norm. |
|  | Afternoon village meeting | ► TC’s drum/sing the six behaviours and pledging song while villagers arrive at meeting site.  ► Opening prayers by the Imam (religious leader) lead prayers for the gathering (Gambian cultural norm).  ► Opening remark by the Alkalo.  ► Introduction of project by PHO.  ► Two dramas (MaaChampion and Funtu) by TCs.  ► Summary of six behaviours from the drama by PHO.  ► Question and answer from village audience led by PHO.  ► Pledge song by TCs.  ► Play ‘Choose soap’ silent animation video [19].  ► Show video of Alkalo (washing hands with soap) and his wife (reheated complementary-food) translated live in local language.  ► Announce MaaChampion competition by PHO.  ► Invite mothers of children aged 6–24 months to pledge to practice behaviours.  ► Give pledged mothers plastic sheets for covering surfaces to enable hygienic drying of utensils/pots on a clean surface.  ► Take a group photo of pledged mothers for the honour board.  ► Closing remark by PHO.  ► More drumming and songs (motivational).  ► Print and display pledged mothers and Alkalo’s photos on honour board at the Bantaba. | Village ‘Bantaba’ (a central place where villagers meet – usually under a large tree) | 4 hours | ► Inform the community/provide instructions.  ► Model or demonstrate behaviour.  ► Engender all motivational drivers, particularly nurture and affiliation.  ► Prompt identification with a role model.  ► Prompt action through pledging.  ► Set graded tasks through competitions.  ► Target mothers for their pledge.  ► Prompt intention formation.  ► Assist memorisation by repetition of song and messages.  ► Display of photos of pledged mothers for contingent reward. |
|  | Community volunteers training | ► Train new assistant MaaSupervisors by village volunteers/trained MaaSupervisors, supervised by PHO. | Village Bantaba | 2 hours | ► Enable encouragement of mothers and competition success.  ► Involve more community members to engender development of social norms. |

| Day 2 | Meeting the Alkalo | ► Greet the Alkalo | Alkalo’s residence | 10 min | As day 1 |
| --- | --- | --- | --- | --- | --- |
|  | Announce to the villagers | As day 1 | As day 1 | 2 hours | As day 1 |
|  | House-to-house visit with MaaSupervisors | ► Engage MaaSupervisors with household visits and boost their confidence.  ► Assess/encourage pledged mothers for progress to next stage. | Residence of each pledged mother | 3 hours | ► Prompt practice of key behaviours.  ► Provide feedback.  ► Prompt self-monitoring/review/community mobilisation. |
|  | Ad hoc women or men meetings held separately in neighbourhoods | ► Glo Germ demonstration.  ► Explain two stories (MaaChampion and Funtu) on flip chart.  ► Play silent animation video ‘Choose soap’ on iPad/laptop. | Neighbourhoods | 30 min | ► Engender disgust through Glo Germ: dirt on hands.  ► Engender all motivational drivers, particularly nurture and affiliation through stories.  ► Engage men and women to support mothers of young children.  ► Prompt specific goal setting. |
| Day  3 | Meeting Alkalo | As day 2 | As day 2 | 20 min | As day 2 |
|  | Announce to the villagers | As day 1 | As day 1 | 2 hours | As day 1 |
|  | House-to-house vist with MaaSupervisors | As day 2. Additionally:  ► During household visits, video mothers who succeeded to became a MaaChampion to show at the afternoon. | As day 2 | 3 hours | As day 2. Additionally:  ► Videoing to provide contingent reward. |
|  | Afternoon village meeting | As day 1. Additionally:  ► Show animation video from India handwashing ‘SuperAmma’ project with spontaneous translation [19].  ► Show videos from mothers who succeeded to become MaaChampions.  ► Take photo of new pledged mothers with their plastic sheets, and of MaaChampions with medals and displayed on honour boards (pledging and certification ceremonies). | As day 1 | 4 hours | As day 1 |
| Day 4 | Meeting Alkalo | As day 2 | As day 2 | 20 min | As day 2 |
|  | Announce to the villagers | As day 1 | As day 1 | 2 hours | As day 1 |
|  | House-to-house vist with MaaSupervisors | As day 3 | As day 3 | 3 hours | As day 3 |
|  | Afternoon village meeting | As day 1. Additionally:  ► Certification ceremony: present medals for MaaChampions/ MaaSupervisors with drumming.  ► Group picture with all MaaChampions, MaaSawar and MaaFamboos for the honour board.  ► During village wide ceremony, erect a complementary-food safety and hygiene board at the village entrance establishing the village as a ‘complementary-food hygiene’ village with drumming/campaign songs and present village certificate to the Alkalo (certification ceremony) for ‘Complementary-food Hygiene Village’ status.  ► Give motivational advice on sustainability by Alkalo, MaaSupervisors and PHOs.  ► Closing remark (emphasis on sustainability). | As day 1 | 4 hours | As day 1. Additionally:  ► Create ownership of the project and self-monitoring to enable sustainability.  ► A community sense of achievement and pride commitment by MaaChampions.  ► Inculcated motivational drivers.  ► Encourage achievement of goals through the board as a reminder. |
| Day 5 | Meeting Alkalo | As day 2 | As day 2 | 20 min | As day 2 |
|  | Announce to the villagers | As day 1 | As day 1 | 2 hours | As day 1 |
|  | House-to-house vist with MaaSupervisors | As day 3 | As day 3 | 3 hours | As day 3 |
|  | Afternoon village meeting | As day 4, but not including erection of the village board or certification. | As day 4 | 4 hours | As day 4 |

PHO= Public Health Officer; TBA=Traditional Birth Attendant; TC=Traditional Communicator; UV= ultraviolet; VHW=Village Health Volunteer
