# Supplementary material for: Effects on childhood infections of promoting safe and hygienic complementary-food handling practices through a community-based programme: A cluster randomised controlled trial in a rural area of The Gambia
Source: PLoS Med. 2021 Jan 11;18(1):e1003260. doi: 10.1371/journal.pmed.1003260 (PMC7799804; doi:10.1371/journal.pmed.1003260)
Supplement: S2 Table — (DOCX) [file pmed.1003260.s010.docx]

**S2 Table. Intervention tools and their application during the intervention.**
(Cited from our former publication [8].)

| **Tool** | **Target population** | **Details** | **Purpose** |
| --- | --- | --- | --- |
| Competitions for mothers and MaaSupervisors | | | |
| Mother’s competitions* | Mother and children <5 years but specifically 6-24 months of age. | All participants are winners. Three stages:  (1) mothers who learnt the six behaviours and pledged to practice the behaviours (MaaFamboo);  (2) mothers who demonstrated during MaaSupervisor and a PHO visit, a community action to encourage a change of social norms;  (3) mothers who did all the above and supported two other mothers to become MaaFamboo (MaaChampion). | To set graded tasks, provide general encouragement (contingent reward) for improved behaviour, prompt identification with a role model and by engaging in sustained practice of six behaviours (MaaSawaar). |
| Maa Supervisors competitions | MaaSupervisors | Older respected woman who must encourage mothers (focus on a minimum of 10) of which 50% must achieve the MaaChampion status. |  |
| Performing arts for all village members. | | | |
| Songs (at times combined with dancing)* | Mother of young children and all villagers attending meetings. | *Campaign song*: information about the six behaviours and benefits of practices and specially explain the benefits of care and love in terms of a grateful child with a successful future.  *Pledged song*: focused on nurture, disgust and purity to encourage mothers to pledge to carry out the practices.  *Welcome song:* a cultural greeting song to welcome and honour the head of the village and those present, with elements of messages added. | To engage communities particularly mothers and to make it easy for mothers to learn the behaviours form the songs. |
| Stories (portrayed in animation and charts)* |  | *Story 1:* story of MaaChampion heard from her grown up child who is now a successful doctor, proudly telling the story to her family.  *Story 2:* story of Funtu about how villagers rejected her and how her child suffered, while meeting the MaaChampion and following her advice made her popular and a good mother. | To stimulate the motivational drivers help mothers drama, understand and remember the behaviours easily.  To communicate the behaviours in a graphically memorable and entertaining way. |
| Drama |  | *One drama:* describing a day in the life of MaaChampion and Funtu. | To prompt identification with the role model (MaaChampion) and consequences of not following the six behaviours. |
| Animations† |  | *Animation 1:* choose soap.^18^ Shows a hand touching faeces and then  eating with and without washing with soap first.  *Animation 2:* SuperAmma.^.18^ Shows a similar story to MaaChampion, but in an Indian village, with reference to handwashing with soap in general rather than references to food safety and hygiene. |  |
| Environmental cues for mothers | | | |
| Posters, danglers and medals* | Mother of young children in the competition. | All had six key intervention practices graphically written on them. The mothers’ posters, dangler and medals were all displayed around the house and kitchen. | To provide non-monetary incentives (contingent reward) for mothers.  To provide visual reminders of the six key messages in the kitchen and household. |
| Plastic sheet |  | A 1.5×1.5 locally available sheet of plastic. | To provide visual reminders of the message about drying pots and utensils on a clean surface.  To facilitate this practice at the start of the programme when villagers do not have easy access to plastic sheets. |
| Other tools for team members or villagers | | | |
| Posters* | All village members. | All had six key intervention behavioural practices graphically written on. | To remind and facilitate the mothers to perform the six key behaviours. |

PHO= Public Health Officer; TBA=Traditional Birth Attendant; TC= Traditional Communicator; UV= ultraviolet; VHW=Village Health Volunteer.

*Tool adapted from Complementary-food safety and hygiene Nepal study [10,11].

†Tool adapted from SuperAmma India Handwashing study [17].
