# Supplementary material for: Effects on childhood infections of promoting safe and hygienic complementary-food handling practices through a community-based programme: A cluster randomised controlled trial in a rural area of The Gambia
Source: PLoS Med. 2021 Jan 11;18(1):e1003260. doi: 10.1371/journal.pmed.1003260 (PMC7799804; doi:10.1371/journal.pmed.1003260)
Supplement: S3 Table — (DOCX) [file pmed.1003260.s011.docx]

**S3 Table. Outcomes and their definitions**

| **Outcome** | **Definition** | **Follow-up measurements (m=month)** | **Source of data** |
| --- | --- | --- | --- |
| Primary outcome:  Composite of 5 complementary-food behaviour outcomes. | Number of times 5 key complementary-food-related behaviours were observed as a proportion of the number of opportunities to perform the behaviours during the observation period (since water-related behaviours were not about food handling, these were not part of the primary outcome). | 6m | Female data collector observed the mother during preparation of breakfast and lunch for the index child. |
| Secondary outcomes: |  |  |  |
| (1) Composite of 5 complementary-food behaviour outcomes. | Number of times 5 key complementary-food-related behaviours were observed (Table 2) as a proportion of the number of opportunities to perform the behaviours during the observation period. (Since water related behaviours were not about food handling, these were not a part of primary outcome). | 32m | Female data collector observed the mother during preparation of breakfast and lunch for the index child. |
| (2) Faecal coliforms (*E. coli*) numbers in complementary-food after preparation before consumption by child, and after storage prior to consumption, and in drinking water; | The log 10 transformation of the count of coliforms (E.Coli) colony count from culturing food and water samples in microbiology lab. | 6m | Food samples collected during the observation of mothers feeding or giving water to the index child. |
| (3) Infant diarrhoea in the past 7 days; | > one-day of diarrhoea (WHO definition: >3 watery stools within 24-hours) in the index child over the previous 7 days as reported by the mother. | 6m  32m | Mother’s report during the questionnaire asked in the home-visit after the observations were complete. |
| (4) Infant acute respiratory infection in the past 7 days; | > one day of acute respiratory infection (ARI) (WHO definition: cough with difficulty breathing) over the previous 7 days as reported by the mother. | 6m  32m | Mother’s report during the questionnaire asked in the home-visit after the observations were complete. |
| (5) Diarrhoea hospital admissions; | Reported hospital admission for the last diarrhoea episode (considered more robust than reports of diarrhoea since hospitalisation is easier for mothers to recall and classify); | 6m  32m | Mother’s report during the questionnaire asked in the home-visit after the observations were complete. |
| (6) ARI hospital admissions; | Reported hospital admission for the last ARI episode (considered more robust than reports of diarrhoea since hospitalisation is easier for mothers to recall and classify). | 6m  32m | Mother’s report during the questionnaire asked in the home-visit after the observations were complete. |
| (7) Boiling child’s drinking-water; | Number of times giving the child cooled boiled water was observed as a proportion of the number of opportunities to perform the behaviour during the observation period. | 6m  32m | Female data collector observed the mother during preparation of breakfast and lunch for the index child. |
| (8) Availability of used soap in the kitchen; | Availability of used soap as observed in the kitchen. | 6m  32m | Female data collector observing the kitchen during home-visit. |
| (9) Availability of used soap in the latrine; | Availability of used soap as observed in the latrine. | 6m  32m | Female data collector observing the kitchen during home-visit. |
