# Supplementary material for: Effects on childhood infections of promoting safe and hygienic complementary-food handling practices through a community-based programme: A cluster randomised controlled trial in a rural area of The Gambia
Source: PLoS Med. 2021 Jan 11;18(1):e1003260. doi: 10.1371/journal.pmed.1003260 (PMC7799804; doi:10.1371/journal.pmed.1003260)
Supplement: S4 Table — (DOCX) [file pmed.1003260.s012.docx]

**S4 Table.** **Baseline characteristics of the villages by intervention allocation.**(Cited from our former publication [8].)

| **Village Characteristics*** | **Control n=15** | **Intervention n=15** |
| --- | --- | --- |
| **Total population** | 5088 | 5219 |
| **Village population,** median [IQR] | 255 [297–400] | 306 [244–352] |
| **Households per village,** median [IQR] | 40 [30–60] | 33 [26–49] |
| **Children aged < 5 years,** median [IQR] | 86 [71–111] | 86 [77–99] |
| **Children aged 6-24 months,** median [IQR] | 39 [34–57] | 43 [33–55] |
| **Major ethnic group in village** Mandingo | 3 (20%) | 5 (33%) |
| Wolof | 5 (33%) | 5 (33%) |
| Fula | 7 (47%) | 5 (33%) |
| **Main income of villages**  Farming | 12 (80%) | 13 (87%) |
| Farming and business | 3 (20%) | 2 (13%) |
| **Distance to nearest health facility**† < 5 km | 6 (40%) | 7 (56%) |
| 5 to ≤ 10 km | 5 (33%) | 4 (27%) |
| > 10 km | 4 (27%) | 4 (27%) |
| **Availability of school in the village** No school | 8 (53%) | 10 (67%) |
| Primary | 5 (33%) | 5 (33%) |
| Primary/middle school | 2 (13%) | 0 |
| **Availability of village/community groups**  Village Development Committee | 15 (100%) | 15 (100%) |
| Water Sub-committee | 11 (73%) | 7 (47%) |
| Women’s Group | 13 (87%) | 15 (100%) |
| **Location of village** North of river | 7 (47%) | 7 (47%) |
| South of river | 8 (53%) | 8 (53%) |
| **Quartile of population site** 1 | 3 (20%) | 3 (20%) |
| 2 | 4 (27%) | 4 (27%) |
| 3 | 4 (27%) | 4 (27%) |
| 4 | 4 (27%) | 4 (27%) |

*Values for the individual variables are numbers (%) or otherwise as stated median [IQR]. Numbers might not add to 100% due to rounding.

†This is the actual travel distance and not the scaled map distance.
