# Supplementary material for: Effects on childhood infections of promoting safe and hygienic complementary-food handling practices through a community-based programme: A cluster randomised controlled trial in a rural area of The Gambia
Source: PLoS Med. 2021 Jan 11;18(1):e1003260. doi: 10.1371/journal.pmed.1003260 (PMC7799804; doi:10.1371/journal.pmed.1003260)
Supplement: S5 Table — (DOCX) [file pmed.1003260.s013.docx]

**S5 Table. Characteristics of mothers in the baseline survey by intervention allocation**(Cited from our former publication [8].)

| Variable / characteristic † | **Control n=300** | **Intervention n=300** |
| --- | --- | --- |
| **Number of children alive for index mother,** median [IQR] | 3 [2-6] | 4 [3-6] |
| **Age group of mother** <20 years | 31 (10%) | 27 (9%) |
| 20-30 years | 177 (59%) | 186 (62%) |
| >30 years | 97 (31%) | 88 (29%) |
| **Education level mother** None/illiterate | 186 (62%) | 138 (46%) |
| Other (Islamic, home etc.) | 56 (19%) | 92 (30%) |
| Primary | 30 (10%) | 39 (13%) |
| Secondary or higher‡ | 28 (9%) | 33 (11%) |
| **Ethnicity of mother** Mandingo | 46 (15%) | 78 (26%) |
| Wolof | 120 (41%) | 96 (32%) |
| Fula | 127 (43%) | 118 (40%) |
| Other | 3 (2%) | 8 (3%) |
| **Occupation of mother§** Farmer | 280 (93%) | 275 (92%) |
| Other\|\| | 20 (7%) | 25 (8%) |
| **Ethnicity of husband** Mandingo | 47 (16%) | 82 (28%) |
| Wolof | 119 (40%) | 96 (33%) |
| Fula | 126 (43%) | 115 (39%) |
| **Structure of house** Cement wall, corrugated roof | 32 (11%) | 43 (15%) |
| Mud wall, corrugated roof | 124 (43%) | 121 (41%) |
| Mud wall, thatched roof | 134 (46%) | 129 (44%) |
| Other | 0 (0%) | 2 (1%) |
| **Belongings** Land | 282 (95%) | 280 (94) |
| Cattle | 173 (58%) | 178 (59%) |
| Goat | 216 (73%) | 216 (72%) |
| Mobile | 253 (85%) | 269 (90%) |
| Radio | 191 (64%) | 203 (68%) |
| Tap | 4 (1%) | 9 (3%) |
| Fridge | 3 (1%) | 8 (3%) |
| **Source of water** Covered well | 119 (40%) | 141 (48%) |
| Open well | 181 (60%) | 152 (52%) |
| **Sex of Index Child** Male | 156 (52%) | 151 (50%) |
| **Age** **of child in months (SD)** | 18 (7.9) | 19 (7.6) |
| **Diarrhoea‡** | 60 (20%) | 82 (28%) |
| **Acute respiratory infection ^#^** | 30 (10%) | 30 (10%) |

† Values for the individual variables are numbers (%) or otherwise as stated median [IQR]. Numbers might not add to 100% due to rounding.

‡Senior secondary or college.

**§**All mothers were housewives, but had additional regular other work.

||Trading, animal husbandry or civil servant.

‡ 3 watery stools in any day in the last 7 days as reported by mother.

**^#^** cough & difficulty breathing in any day in the last 7 day as reported by mother.
