# Supplementary material for: Effects on childhood infections of promoting safe and hygienic complementary-food handling practices through a community-based programme: A cluster randomised controlled trial in a rural area of The Gambia
Source: PLoS Med. 2021 Jan 11;18(1):e1003260. doi: 10.1371/journal.pmed.1003260 (PMC7799804; doi:10.1371/journal.pmed.1003260)
Supplement: S6 Table — (DOCX) [file pmed.1003260.s014.docx]

**S6 Table. Effect of intervention on outcomes with a baseline data available and adjusted for baseline data.**

| **Health outcomes or process measures** | 6 month | | | | 32 month | | | |
| --- | --- | --- | --- | --- | --- | --- | --- | --- |
|  | **Control n=308** | **Intervention n=307** | **Partially* Adjusted** | **Fully** Adjusted** | **Control n=377** | **Intervention n=370** | **Partially* Adjusted** | **Fully** Adjusted** |
|  | **n (%)** | **n (%)** | **RR**  **(95%CI) P-value** | **RR**  **(95%CI) P-value** | **n (%)** | **n (%)** | **RR**  **(95%CI) P-value** | **RR**  **(95%CI) P-value** |
| Diarrhoea | 202 (66%) | 90  (26%) | 0.39 (0.33,0.48) | 0.40 (0.32,0.49) | 102 (27%) | 69  (19%) | 0.64  (0.45, 0.90) | 0.65 (0.45,0.93) |
|  |  |  | <0.001 | <0.001 |  |  | 0.011 | 0.019 |
| Acute respiratory tract infection | 129 (42%) | 86  (28%) | 0.68  (0.53,0.86) | 0.70 (0.55,0.89) | 77 (20%) | 55  (15%) | 0.74 (0.48,1.15) | 0.79  (0.52,1.20) |
|  |  |  | 0.001 | 0.003 |  |  | 0.182 | 0.269 |
| RR: Risk Ratio.  * Adjusted for cluster level covariates used in the randomisation (location of village and village size) and cluster level baseline rate of the outcome.  ** Adjusted for cluster level covariates used in the randomisation (location of village and village size), cluster level baseline rate of the outcome, and individual level covariates (mothers age, mothers education, gender, number of children in household). | | | | | | | | |
