# Supplementary material for: Effects on childhood infections of promoting safe and hygienic complementary-food handling practices through a community-based programme: A cluster randomised controlled trial in a rural area of The Gambia
Source: PLoS Med. 2021 Jan 11;18(1):e1003260. doi: 10.1371/journal.pmed.1003260 (PMC7799804; doi:10.1371/journal.pmed.1003260)
Supplement: S7 Table — (DOCX) [file pmed.1003260.s015.docx]

**S7 Table. The effect of intervention on the opportunities to practice the key behaviours promoted through the intervention presented by ‘new mothers’ and those who were at least pregnant or had their babies during the intervention team activities.**

|  | **Mothers pregnant or with infant >26 months at  intervention delivery** | | | | | | | | | **New mothers with children <=26 months** | | | | | | | | | **Interaction test** | | |
| --- | --- | --- | --- | --- | --- | --- | --- | --- | --- | --- | --- | --- | --- | --- | --- | --- | --- | --- | --- | --- | --- |
| **Outcomes/**  **behaviours** | **Number of correct behaviours (events)** | | **Number of opportunities for doing the behaviour** | | **Incidence rate** | | **Unadjusted IRR**  **(95% CI)p-value** | **Adjusted IRR**  **(95% CI) †p-value** | **Fully adjusted IRR**  **(95% CI) †p-value** | **Number of correct behaviours (events)** | **Number of opportunities for doing the behaviour** | | **Incidence rate** | | **Unadjusted IRR**  **(95% CI)p-value** | | **Adjusted IRR**  **(95% CI) †p-value** | **Fully adjusted IRR**  **(95% CI) †p-value** | **Unadjusted** | **Adjusted** | **Fully adjusted** |
|  | C | I | C | I | C | I |  |  |  | C | I | C | I | C | I |  |  |  |  |  |  |
| Five key behave-iours‡ | 620 | 633 | 1602 | 1377 | 0.39 | 0.45 | 1.18  (1.04, 1.35)  0.013 | 1.19  (1.04, 1.36)  0.011 | 1.19  (1.04, 1.36)  0.014 | 1082 | 1286 | 2653 | 2744 | 0.408 | 0.469 | 1.15  (1.02, 1.30)  0.018 | 1.16  (1.03, 1.29)  0.01 | 1.16  (1.04, 1.30)  0.008 | 0.624 | 0.624 | 0.742 |
| Hand-washing^#^ before cooking | 43 | 60 | 194 | 174 | 0.22 | 0.34 | 1.56  (1.05, 2.30)  0.027 | 1.64  (1.10, 2.44)  0.015 | 1.53  (0.99, 2.35)  0.056 | 76 | 121 | 319 | 330 | 0.23 | 0.36 | 1.54  (1.16, 2.05)  0.003 | 1.56  (1.17, 2.08)  0.003 | 1.53  (1.13, 2.07)  0.005 | 0.965 | 0.930 | 0.925 |
| Hand-washing during cooking | 29 | 40 | 420 | 333 | 0.06 | 0.12 | 1.80 (1.02, 3.18) 0.042 | 1.74 (1.02, 2.92) 0.034 | 1.74 (1.02, 2.98) 0.044 | 33 | 80 | 592 | 608 | 0.05 | 0.13 | 2.36 (1.57, 3.54) < 0.001 | 2.35 (1.57, 3.53) < 0.001 | 2.28 (1.49, 3.48) < 0.001 | 0.332 | 0.33 | 0.338 |
| Hand- washing^#^ before feeding child | 11 | 25 | 263 | 228 | 0.04 | 0.10 | 2.64 (1.24, 5.65) 0.012 | 2.67 (1.22, 5.83)  0.014 | 2.35  (1.01,  5.44)  0.047 | 21 | 42 | 465 | 487 | 0.04 | 0.08 | 1.97  (1.04, 3.74) 0.038 | 2.01 (1.06, 3.81) 0.032 | 1.82 (0.90, 3.69)  0.095 | 0.487 | 0.49 | 0.518 |
| Wash-ing pots and utensils & drying on clean surface | 508 | 485 | 664 | 593 | 0.77 | 0.81 | 1.07  (0.93, 1.23)  0.338 | 1.07  (0.93, 1.23)  0.324 | 1.08  (0.94, 1.25)  0.297 | 889 | 978 | 1148 | 1192 | 0.77 | 0.82 | 1.06  (0.95, 1.18)  0.302 | 1.06  (0.96, 1.18)  0.246 | 1.07  (0.97, 1.19)  0.18 | 0.833 | 0.840 | 0.918 |
| Re-heating comple-mentary-food before feeding | 29 | 23 | 61 | 49 | 0.48 | 0.46 | 0.99  (0.57, 1.71)  0.964 | 0.99  (0.57, 1.71)  0.962 | 1.05  (0.57, 1.91)  0.884 | 63 | 65 | 129 | 127 | 0.48 | 0.51 | 1.05  (0.74, 1.48) 0.791 | 1.09  (0.77, 1.55)  0.64 | 1.04  (0.72, 1.50)  0.839 | 0.857 | 0.767 | 0.844 |
| Boiling child’s drinking water | 3 | 31 | 163 | 152 | 0.02 | 0.20 | 13.3  (2.9, 60.5)  0.001 | 14.8  (2.9, 75.6)  0.001 | 12.8  (2.9, 56.1)  0.001 | 6 | 136 | 285 | 320 | 0.02 | 0.42 | 21.5  (7.8, 59.1)  <0.001 | 21.4  (8.3, 55.00)  <0.001 | 26.4  (9.2, 76.1)  <0.001 | 0.453 | 0.456 | 0.278 |
|  | | | | | | | | | | | | | | | | | | | | | |

C = control clusters/villages; I = intervention clusters/villages; IRR=incident rate ratio.

* CI, Confidence interval; IRR, incidence rate ratio.

†Adjusted for cluster level covariates used in the randomisation (location (north or south of the river), and village size).

‡ Primary outcome: The five key practices were: 1. handwashing with soap and water before food preparation/cooking, 2. washing of pots and utensils before cooking and/or serving food, 3. handwashing with soap and water during food preparation/cooking if hands became contaminated, 4. handwashing with soap and water before feeding child, and 5. reheating stored complementary-food before second feeding to the child.

^#^ All handwashing was with soap.
