# Supplementary material for: Effects on childhood infections of promoting safe and hygienic complementary-food handling practices through a community-based programme: A cluster randomised controlled trial in a rural area of The Gambia
Source: PLoS Med. 2021 Jan 11;18(1):e1003260. doi: 10.1371/journal.pmed.1003260 (PMC7799804; doi:10.1371/journal.pmed.1003260)
Supplement: S8 Table — (DOCX) [file pmed.1003260.s016.docx]

**S8 Table. Effect of intervention on outcomes other than observed complementary-food safety and hygiene behaviours presented by ‘new mothers’ and those who were at least pregnant or had their babies during the intervention team activities.**

|  | **>26 months** | | | | | **<=26 months** | | | | | **Interaction test** | | | |
| --- | --- | --- | --- | --- | --- | --- | --- | --- | --- | --- | --- | --- | --- | --- |
| **Health outcomes or process measures** | **Control n=**  **136** | **Intervention n=**  **117** | **Unadjusted RR**  **(95% CI)** | **Adjusted RR**  **(95% CI) †** | **Fully Adjusted RR**  **(95% CI) †** | **Control n=**  **242** | **Intervention n=**  **253** | **Unadjusted RR**  **(95% CI)** | **Adjusted RR**  **(95% CI) †** | **Fully Adjusted RR (95% CI) †** | **Unadjusted** | **Adjusted** | **Fully Adjusted** |  |
|  | **n (%)** | **n (%)** | **p-value** | **p-value** | **p-value** | **n (%)** | **n (%)** | **p-value** | **p-value** | **p-value** |  |  |  |  |
| Admissions for diarrhoea ‡ | 6  (7) | 1  (1) | 0.22  (0.02, 1.41)  0.175 | 0.17  (0.12, 1.09)  0.059 | 0.22  (0.13, 1.08)  0.058 | 9  (5) | 4 (3) | 0.50  (0.19, 1.03)  0.061 | 0.48  (0.19, 1.00)  0.049 | 0.35  (0.12, 1.01)  0.053 | 0.541 | 0.538 | 0.658 |  |
| Diarrhoea‡‡ | 40 (29) | 20 (17) | 0.58  (0.34, 1.00) | 0.59  (0.34, 0.99) | 0.61  (0.34, 1.02) | 62 (26) | 49 (19) | 0.74  (0.53, 1.05) | 0.74  (0.58, 1.02) | 0.71  (0.49, 1.01) | 0.441 | 0.476 | 0.704 |  |
|  |  |  | 0.049 | 0.048 | 0.058 |  |  | 0.091 | 0.051 | 0.052 |  |  |  |  |
| Admission for Acute respiratory tract infection | 3 (8) | 3 (8) | 1.08  (0.26, 4.56) | 1.08  (0.16, 7.22) | 1.02  (0.13, 7.78) | 3 (3) | 4 (5) | 1.22  (0.72, 14.70) | 1.17  (0.72, 14.43) | 1.10  (0.46, 19.74) | 0.245 | 0.282 | 0.279 |  |
|  |  |  | 0.913 | 0.934 | 0.986 |  |  | 0.103 | 0.104 | 0.2 |  |  |  |  |
| Acute respiratory tract infection^#^ | 22 (16) | 18 (15) | 0.91  (0.40, 2.06) | 0.91  (0.41, 2.03) | 1.14  (0.55, 2.35) | 55 (23) | 37 (15) | 0.64  (0.41, 0.99) | 0.69  (0.42, 0.96) | 0.64  (0.42, 0.98) | 0.057 | 0.044 | 0.07 |  |
|  |  |  | 0.813) | 0.815 | 0.717 |  |  | 0.043 | 0.033 | 0.041 |  |  |  |  |
| Observe soap available at kitchen†† | 89 (65) | 84 (72) | 1.11  (0.67, 1.21) | 1.13  (0.79, 1.01) | 1.12  (0.77, 1.03) | 148 (62) | 176 (74) | 1.18  (1.01, 1.38) | 1.19  (1.02, 1.38) | 1.12  (0.99, 1.43) | 0.625 | 0.60 | 0.452 |  |
|  |  |  | 0.083 | 0.058 | 0.058 |  |  | 0.039 | 0.024 | 0.052 |  |  |  |  |
| Observed soap available at pit latrine†† | 54 (40) | 64 (55) | 1.38  (1.06, 1.80) | 1.39  (1.08, 1.80) | 1.35  (1.02, 1.79) | 99 (42) | 133 (55) | 1.32  (1.00, 1.73) | 1.35  (1.08, 1.68) | 1.28  (1.02, 1.61) | 0.781 | 0.801 | 0.724 |  |
|  |  |  | 0.018 | 0.012 | 0.033 |  |  | 0.051 | 0.008 | 0.033 |  |  |  |  |

* CI=confidence interval; MD=mean difference; RR= Relative risk.

† Adjusted for cluster level covariates used in the randomisation (location (north or south of the river), and village size).

†F Adjusted for mothers age, mothers’ education level, gender, number of children in the household, and cluster level covariates used in the randomisation (location (north or south of the river), and village size).

‡ Child hospital admission during the last diarrhoea episode as reported by mother.

‡‡ 3 watery stools in any day in the last 7 days as reported by mother.

# Cough & difficulty breathing in any day in the last 7 day as reported by mother.

†† There were no explicit messages given regarding these variables during the complementary-food safety and hygiene intervention
